# Antisense oligonucleotide allele-specific targeting of EFEMP1 in a patient-derived model of Doyne honeycomb retinal dystrophy

**DOI:** 10.1016/j.omtn.2026.102955

**Published:** 2026-05-19

**Authors:** Farah O. Rezek, Beatriz Sanchez-Pintado, Emily R. Eden, Julio C. Corral-Serrano, Nancy Aychoua, Andrew R. Webster, Thales A.C. de Guimarães, Amanda-Jayne F. Carr, Michel Michaelides, Michael E. Cheetham, Jacqueline van der Spuy

**Affiliations:** 1 UCL Institute of Ophthalmology, University College London, London EC1V 9EL, UK; 2 Moorfields Eye Hospital NHS Foundation Trust, London, UK

**Keywords:** MT: oligonucleotides: therapies and applications, Doyne honeycomb retinal dystrophy, EFEMP1, inherited macular degeneration, antisense oligonucleotide, retinal pigment epithelium, drusen, induced pluripotent stem cell, extracellular matrix, apolipoprotein E, collagen IV

## Abstract

Doyne honeycomb retinal dystrophy is an incurable juvenile macular dystrophy that leads to visual impairment by early to mid-adulthood. It is an autosomal dominant disorder caused by a c.1033C>T, p.(Arg345Trp) variant in *EFEMP1*, and is characterized by the early onset of extracellular deposition of drusen between the retinal pigment epithelium and underlying Bruch’s membrane. In this study, we developed an antisense oligonucleotide approach to target *EFEMP1*. We reprogrammed patient-derived renal epithelial cells to induced pluripotent stem cells, followed by directed differentiation to retinal pigment epithelium and compared the phenotype to gene-corrected and *EFEMP1* knockout patient-derived retinal pigment epithelium. In the patient-derived disease model, remodeling of the extracellular matrix (ECM) occurred with progressive accumulation of the drusen-associated proteins apolipoprotein E and collagen IV, in addition to the intracellular accumulation and extracellular deposition of lipids. We developed an allele-specific antisense oligonucleotide which specifically and effectively promoted the clearance of the *EFEMP1* c.1033C>T transcript in the patient-derived disease model following assisted or gymnotic delivery. Gymnotic delivery rescued remodeling of the ECM, reduced intracellular accumulation of lipids, and cleared extracellular deposits, even after the onset of the disease phenotype, suggesting that this could be a practical and effective therapeutic approach.

## Graphical abstract

**Figure:**
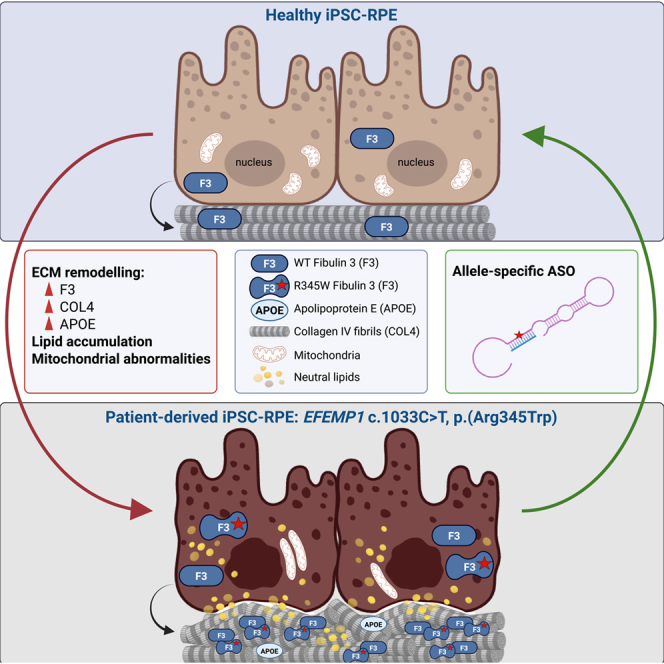

## Teaser

Rezek and colleagues demonstrate that an ASO targeting the genetic cause of an autosomal dominant juvenile macular dystrophy leading to visual loss results in effective and specific knockdown of the disease-associated transcript and amelioration of the disease phenotype in a patient-derived model, providing evidence for a promising therapeutic strategy.

## Introduction

Doyne honeycomb retinal dystrophy (DHRD) is a progressive and incurable juvenile macular dystrophy that is characterized by the early onset of radial deposition of peripapillary and macular drusen that merge over time in a “honeycomb” pattern.^1^^,^^2^ The lipoproteinaceous drusen are deposited extracellularly between the retinal pigment epithelium (RPE) basement membrane and the underlying layers of Bruch’s membrane. Though the onset of visual symptoms varies, DHRD typically leads to visual impairment in early to mid-adulthood. However, extensive drusen can be present as early as the second decade of life.^1^^,^^2^ Early visual symptoms include decreased visual acuity, photophobia, metamorphopsia, dyschromatopsia, and relative scotomas that progress to the atrophy of the RPE leading to central visual loss. A worse prognosis is associated with choroidal neovascularization (CNV).^3^

DHRD is an autosomal dominant disorder caused by the c.1033C>T, p.(Arg345Trp) variant in the *epidermal growth factor (EGF)-containing fibulin-like extracellular matrix (ECM) protein 1* (*EFEMP1*) gene.^4^ The EFEMP1, or fibulin 3 (F3), protein is an extracellular glycoprotein that is synthesized by the RPE and secreted into the collagen IV-rich RPE basement membrane, where it contributes to the structural integrity of the ECM. The Arg345Trp substitution is in the final Ca^2+^ binding EGF domain of EFEMP1, which alters disulfide bond formation and the structural integrity of the protein, resulting in increased stability and accumulation of the mutant protein in the ECM.^5^^,^^6^^,^^7^^,^^8^ Extracellular accumulation of EFEMP1 Arg345Trp also causes increased expression of its binding partner, tissue inhibitor of metalloproteinase 3 (TIMP3), resulting in reduced activity of matrix metalloproteinases (MMP), such as MMP2 and MMP9, and creating an imbalance that results in the accumulation of ECM aggregates.^9^^,^^10^ The remodeling process triggers an interplay that increases levels of complement component 3 (C3) and complement factor B (CFB) that exacerbate the ECM remodeling and sub-RPE deposition.^11^^,^^12^^,^^13^ Moreover, EFEMP1 Arg345Trp has been shown to exert a hyper-inhibitory effect on epidermal growth factor receptor (EGFR) signaling, potentially through the deactivation of the PI3K/AKT pathway. This disruption leads to reduced expression of carboxylesterase 1 (CES1), impairing cholesterol efflux and resulting in abnormal lipid accumulation within the RPE cells and the basement membrane.^14^ These pathological changes collectively disrupt the structural and functional integrity of the RPE basement membrane, leading to the deposition of drusen containing components, such as apolipoprotein E (APOE), C3, and TIMP3. Increased accumulation of sub-RPE basal deposits rich in lipids and proteins, including APOE, TIMP3, and EFEMP1, has been reported in a patient-derived DHRD RPE disease model.^13^

Patients who harbor biallelic loss-of-function *EFEMP1* variants develop a pronounced connective tissue disease characterized by multiple and recurrent abdominal and thoracic herniae, myopia, hypermobile joints, scoliosis, and thin translucent skin; however, macular dystrophy is absent in these patients.^15^ Moreover, macular dystrophy is absent in *Efemp1* knockout mice, and the knockout of *Efemp1* has been shown to be protective against the development of sub-RPE deposits.^16^^,^^17^ These data support an EFEMP1 Arg345Trp toxic gain of function and the absence of haploinsufficiency in DHRD.

Therefore, in this study, we designed an allele-specific antisense oligonucleotide (ASO) therapy to induce the knockdown of c.1033C>T *EFEMP1* expression in a patient-derived disease model of DHRD. We reprogrammed patient renal epithelial cells to induced pluripotent stem cells (iPSCs), followed by directed differentiation to RPE (iRPE). Our patient iRPE model faithfully recapitulated the disease phenotype, including the accumulation of lipids, remodeling of the ECM, and the sub-RPE deposition of drusen-associated components. The treatment of patient-derived iRPE with therapeutic ASO resulted in allele-specific targeting of *EFEMP1* c.1033C>T transcript and efficient resolution of pathogenic changes, even after the onset of the disease phenotype *in vitro*.

## Results

### DHRD patient clinical phenotype

Renal epithelial cells were purified from the urine of a 60-year-old male patient clinically diagnosed with DHRD and confirmed to harbor the autosomal dominant *EFEMP1* variant c.1033C>T, p.(Arg345Trp). The patient was first diagnosed with DHRD in his early twenties, presymptomatically, following retinal examination due to the family history. Ultra-widefield fundus images (Optos scanning laser ophthalmoscope) and autofluorescence images showed hyperautofluorescent deposits at the macula, some with a radial configuration, with deposits directly adjacent to the optic disc (Figure S1). Heidelberg Spectralis optical coherence tomography (OCT) showed an extensive, hyperreflective deposit underlying the neurosensory retina, greater at the left macula than the right (Figure S1).

### Correction of *EFEMP1* c.1033C>T and *EFEMP1* knockout in patient iPSC

The DHRD patient renal epithelial cells were reprogrammed to iPSCs, as described previously.^18^^,^^19^ CRISPR-Cas9 homology-directed repair (HDR) using *in vitro* assembled *EFEMP1* CRISPR RNA/t*rans*-activating CRISPR RNA (crRNA/tracrRNA)-Cas9 ribonucleoprotein (RNP) together with a single-stranded oligonucleotide (ssODN) repair template was used to correct the c.1033C>T, p.(Arg345Trp) variant in the patient iPSC line, thereby generating an isogenic control (Figure S2A). In addition, the ssODN repair template introduced a heterozygous synonymous change in the protospacer adjacent motif (PAM). CRIPSR-Cas9 non-homologous end joining (NHEJ) targeting *EFEMP1* exon 5 introduced a homozygous insertion of a single nucleotide (G), inducing a frameshift and premature translation termination of the *EFEMP1* transcript (c.215_216insG, p.(Lys73GlufsX81)) (Figure S2B). crRNA and ssODN sequences are listed in Tables S1 and S2. No off-target editing was observed in the isogenic lines at the top 10 predicted off-target genomic loci (Table S3). iPSC cultures from the DHRD patient iPSC (R345W), isogenic repaired iPSC (ISOCON), and isogenic knockout (ISOKO) lines uniformly expressed pluripotency markers (*OCT4*, *NANOG*, *TRA1-181*, and *SSEA4*) (Figure S2C).

### Phenotypic characteristics of patient iPSC-iRPE

**Figure 1:**
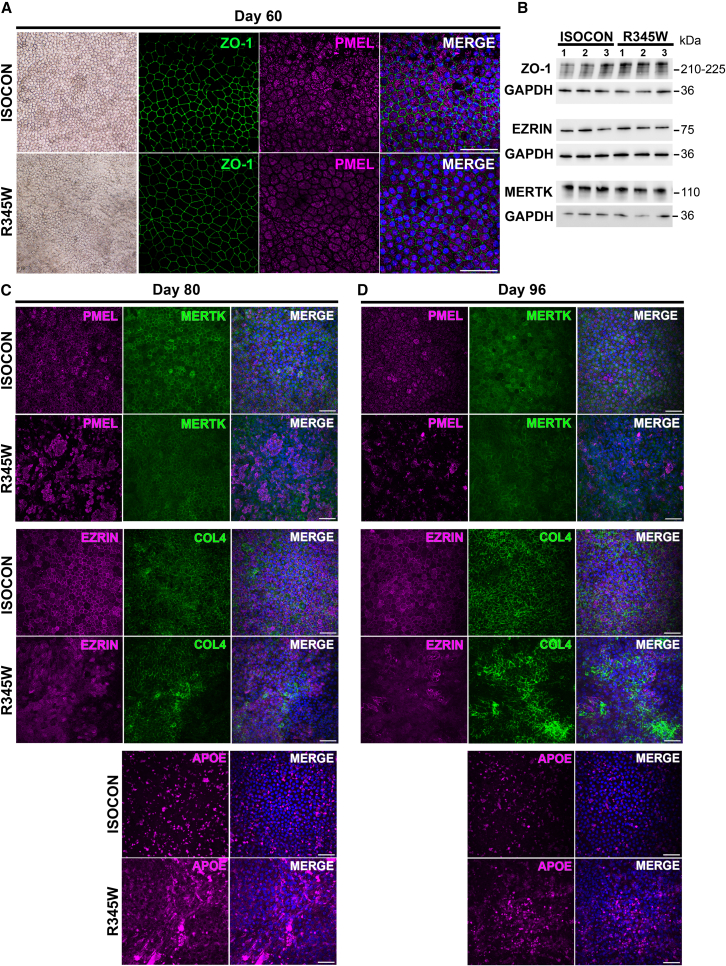
Phenotypic characterization of isogenic and R345W iRPE (A) Bright field microscopy and IF of iRPE from ISOCON and R345W iRPE at day 60 of differentiation, showing pigmented cobblestone morphology, tight junction formation (ZO-1, green), and pre-melanosome (PMEL, magenta) protein expression. Nuclei (blue) are labeled with DAPI. Scale bars, 50 μm. (B) Western blot analysis of protein extracts from ISOCON and R345W iRPE cell lysates for ZO-1, Ezrin, and MERTK. (C) IF of ISOCON and R345W iRPE at day 80 of differentiation and (D) at day 96 of differentiation to detect PMEL (magenta) co-labeled with MERTK (green), Ezrin (magenta) co-labeled with COL4 (green), and APOE (magenta). Nuclei (blue) are labeled with DAPI. Scale bars, 50 μm.

iPSC lines were differentiated to RPE (iRPE) by the method described in Regent et al.^20^ Light microscopy images of the isogenic control (ISOCON) and R345W patient line at day 60 of RPE differentiation (Figure 1A) demonstrated a typical RPE cobblestone morphology, with pronounced cell borders. The ISOCON iRPE formed a homogenous monolayer, while the R345W iRPE displayed a more heterogeneous morphology. Immunofluorescence (IF) analysis of both iRPE lines demonstrated clear expression of the tight junction protein ZO-1, which localized to the cell-cell borders in a continuous, hexagonal pattern consistent with the formation of an epithelial monolayer with tight junctions (Figure 1A). PMEL, a marker of melanosome biogenesis, was also detected in both lines, appearing as intracellular puncta indicative of developing pigment organelles (Figure 1A). These data confirm the expression of key RPE markers associated with epithelial identity and early melanosome formation. Western blot analyses of both lines demonstrated robust expression of the RPE characteristic markers ZO-1, Ezrin, and MERTK at day 110 of differentiation (Figure 1B).

At day 80 and day 96, immunostaining of the ISOCON and R345W iRPE revealed phenotypic differences in the pattern and localization of key RPE markers (Figure 1C). The R345W iRPE displayed a markedly patchy and heterogeneous PMEL distribution, with areas of diminished signal interspersed among irregularly distributed PMEL-positive regions, potentially indicating a delay in iRPE maturity relative to the ISOCON iRPE. MERTK immunostaining was primarily localized to the plasma membrane as expected and illustrated the differences in cell morphology between the ISOCON and R345W iRPE at both time points, with a less organized and more heterogeneous cell population apparent in the R345W line, that deteriorated between days 80 and 96. Immunostaining of collagen IV (COL4), a marker of the basement membrane, exhibited an evenly distributed and homogenous pattern, reflecting a well-organized basement membrane in ISOCON iRPE. Conversely, the COL4 immunolocalization in the R345W iRPE exhibited an uneven pattern, with bright accumulations, reflecting a disrupted basement membrane. Similarly, Ezrin immunolocalization, which marks the apical microvilli, was uniform in the ISOCON line but appeared patchy and irregular in the R345W line. APOE deposits were observed in both the ISOCON and R345W iRPE at days 80 and 96 time points; however, deposits were larger and more extensively distributed in the R345W line, suggesting that this substitution leads to increased APOE accumulation, a major component of drusen formation. These results collectively showed that both the ISOCON and R345W iPSC lines could form RPE, as evidenced by the expression of hallmark RPE markers. However, the presence of the R345W variant results in phenotypic alterations, suggesting that while fundamental RPE differentiation is preserved, the variant disrupts the structural organization of the ECM components, potentially compromising RPE function and contributing to disease pathology.

### Apicobasal characteristics of patient iRPE

**Figure 2:**
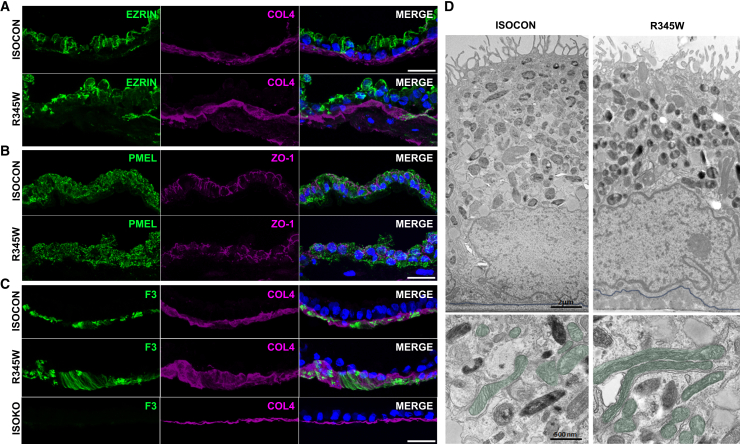
Phenotypic abnormalities are exacerbated in R345W iRPE over time (A) IF of transverse sections (day 98 of differentiation) of ISOCON and R345W iRPE for apical marker Ezrin (green) and basement layer marker COL4 (magenta). Nuclei (blue) are labeled with DAPI. Scale bars, 25 μm. (B) IF of transverse sections (day 98 of differentiation) of ISOCON and R345W iRPE for PMEL (green) and ZO-1 (magenta). Nuclei (blue) are labeled with DAPI. Scale bars, 25 μm. (C) IF of transverse sections (day 98 of differentiation) of ISOCON, R345W, and ISOKO iRPE for EFEMP1 (F3) (green) and COL4 (magenta). Nuclei (blue) are labeled with DAPI. Scale bars, 25 μm. (D) Upper: representative TEM images showing characteristic morphology of polarized pigmented RPE in ISOCON and R345W iRPE. Basal deposits between the basal membrane and the underlying transwell membrane in R345W iRPE are outlined in blue. Scale bars, 2 μm. Lower: representative TEM images showing altered mitochondria morphology (false-colored green) in R345W iRPE. Scale bars, 500 nm.

To characterize the iRPE and confirm their apicobasal polarity, day 98 vertically embedded cross sections were immunostained for RPE markers (Figure 2). The ISOCON iRPE formed a well-organized, uniform monolayer. Ezrin was appropriately localized to the apical surface, with COL4 immunolocalization marking a continuous and uniform basal layer (Figure 2A). In contrast, the R345W iRPE displayed a disorganized phenotype, with a dysmorphic basal membrane and the cells failing to form a uniform monolayer. COL4 immunolocalization revealed a disrupted and thickened basement membrane, indicating abnormalities in structural integrity (Figure 2A). Distribution of PMEL localization was comparable between the ISOCON and R345W iRPE, although ZO-1 localization at cell-cell junctions appeared more fragmented and discontinuous in the R345W line relative to the control, potentially indicating a disruption of tight junctions in these cells (Figure 2B). IF for EFEMP1 (fibulin 3 [F3]) was conducted in ISOCON, R345W, and ISOKO iRPE (Figure 2C). ISOCON iRPE exhibited a clustered distribution of F3 across the basal layer, with the R345W iRPE also demonstrating a similar pattern but with the appearance and accumulation of more filamentous-shaped F3. Immunolocalization of F3 in the ISOKO iRPE demonstrated both that *EFEMP1* expression was successfully knocked out of this cell line and that the antibody used was specific to F3, as no signal was observed. Moreover, COL4 immunolocalization in the ISOKO iRPE was similar to that in the ISOCON iRPE, demarcating a continuous and well-organized basement membrane (Figure 2C).

The ultrastructure of ISOCON and R345W iRPE was examined by transmission electron microscopy (TEM) at day 158 of differentiation (Figure 2D). Both ISOCON and R345W iRPE demonstrated a typical polarized RPE morphology with basally located nuclei, numerous elongated microvilli extending from the apical surface, and cytoplasm rich in melanin granules concentrated apically (Figure 2D, upper). However, basal deposits (blue outline) were visible between the basement membrane and the underlying transwell membrane in patient-derived iRPE cells (Figure 2D, upper). Additionally, both ISOCON and R345W iRPE contained abundant mitochondria. However, altered morphology of mitochondria (false-colored green), which were sometimes elongated, were observed in the R345W iRPE with the cristae often appearing to be orientated longitudinally (Figure 2D, lower), raising the possibility of mitochondrial dysfunction as a feature of the disease pathology.

### *In vitro* screen of antisense oligonucleotides targeting *EFEMP1* c.1033C>T

**Figure 3:**
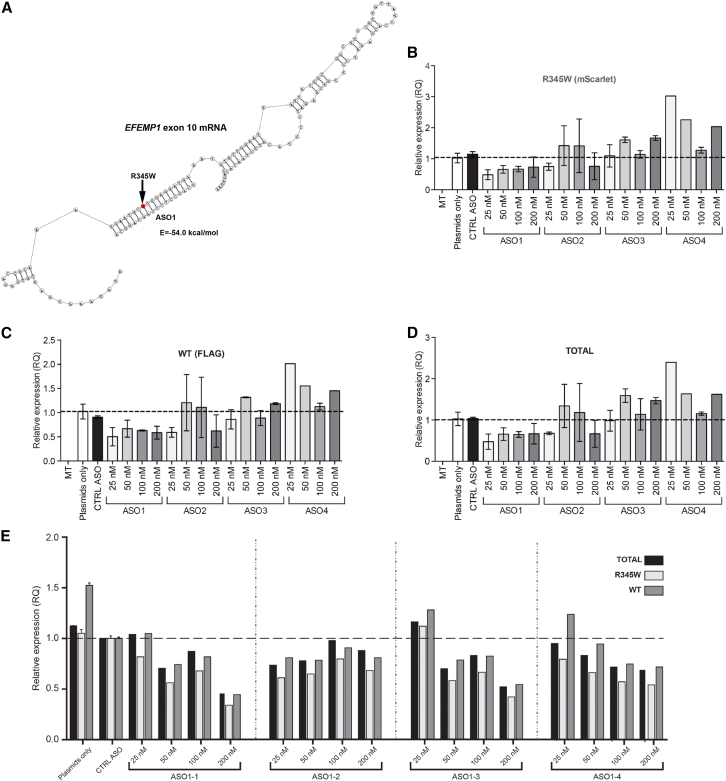
*In vitro* screen of *EFEMP1*-targeting ASO in HEK293T cells (A) *In silico* analysis of *EFEMP1* (exon 10 mRNA) (RNAstructure, University of Rochester). The c.1033C>T, p.(Arg345Trp) variant is highlighted by an arrow and the binding of ASO1 is shown. The calculated difference in free energy between the target sequence alone (lowest free energy value of −21.9 kcal/mol) and the ASO1-bound target sequence (−54.0 kcal/mol) is >21 kcal/mol, meeting the requirements for stable ASO targeting.^21^ (B–D) qPCR analysis of R345W *EFEMP1* (B), WT *EFEMP1* (C), and total *EFEMP1* (D) transcript levels following transfection of HEK293T cells with pEFEMP1(R345W)-mScarlet and pEFEMP1(WT)-3×FLAG together with control ASO (CTRL) (50 nM) or increasing amounts (25, 50, 100, and 200 nM) of ASO1, ASO2, ASO3, and ASO4. *EFEMP1* transcript levels are expressed relative to the untreated “plasmid only” condition. No expression of *EFEMP1* was detected in cells that were treated with the transfection reagent only (MT). The experiment was repeated three times (*N* = 3) with four technical replicates (*n* = 4) per condition in each experiment. Bars represent mean ± SD. (E) qPCR analysis of total (black bars), R345W (light gray bars), and WT (dark gray bars) *EFEMP1* transcript levels following the transfection of HEK293T cells with pEFEMP1(R345W)-mScarlet and pEFEMP1(WT)-3×FLAG together with control ASO (CTRL) (50 nM) or increasing amounts (25, 50, 100, and 200 nM) of ASO1.1, ASO1.2, ASO1.3, and ASO1.4. *EFEMP1* transcript levels are expressed relative to the untreated “plasmid only” condition. The experiment was repeated three times (*N* = 3) with four technical replicates (*n* = 4) per condition in each experiment. Bars represent mean ± SD.

Four 18-mer ASO (ASO1–4) spanning the c.1033C>T variation in *EFEMP1* (Table S4) were designed to induce RNase H1-mediated degradation of the *EFEMP1* target mRNA and analyzed *in silico*, as described previously.^21^ The thermodynamic binding stability of each ASO sequence was analyzed *in silico* using a 124 bp sequence comprising exon 10 of R345W *EFEMP1* as target RNA (RNAstructure, University of Rochester) to determine the difference in free energy of the target sequence alone in comparison to the ASO-bound target sequence, as shown for ASO1 (Figure 3A). This analysis confirmed the stable binding of all ASOs to the target sequence. An *in vitro* screening of ASO1–4 in HEK293T cells was conducted to determine their ability to knockdown *EFEMP1* (Figures 3B–3E). HEK293T cells co-transfected with R345W EFEMP1-mScarlet and wild-type (WT) EFEMP1-FLAG plasmids were treated with control ASO (CTRL) (50 nM) or increasing concentrations (25, 50, 100, and 200 nM) of ASO1, 2, 3, or 4. Quantitation of R345W *EFEMP1* (Figure 3B), WT *EFEMP1* (Figure 3C), and total *EFEMP1* (Figure 3D) transcripts by quantitative PCR (qPCR) showed a reduction in all *EFEMP1* levels (R345W, WT, and TOTAL) compared to the CTRL ASO and untreated cells (plasmids only), only with ASO1 (Figure 3). Reduced levels of *EFEMP1* (R345W, WT, total) with ASO2 were evident only at the highest and lowest concentrations. No reduction of *EFEMP1* R345W, WT, or total transcript levels was evident with ASO3 or with ASO4. No expression of *EFEMP1* was detected in cells that were mock transfected (MT). Four derivatives of the best-preforming ASO1 (ASO1.1–1.4) (Table S4) were tested *in vitro* in HEK293T cells (Figure 3E). Levels of *EFEMP1* (total, R345W, and WT) were measured by qPCR. All ASO, except for ASO1.2, showed a concentration-dependent decline in *EFEMP1* (total, R345W, and WT) transcript levels compared to the untreated (plasmid only) and control ASO (CTRL) conditions, and the highest level of *EFEMP1* reduction was achieved with 200 nM ASO1.1. While ASO1.2 could effectively reduce *EFEMP1* levels at all concentrations tested, the ASO1.2-mediated reduction in *EFEMP1* levels was not concentration-dependent. All ASO (1.1–1.4) mediated a greater reduction in R345W *EFEMP1* compared to WT *EFEMP1* (Figure 3E).

### Allele-specific *EFEMP1* targeting following ASO transient transfection of iRPE

**Figure 4:**
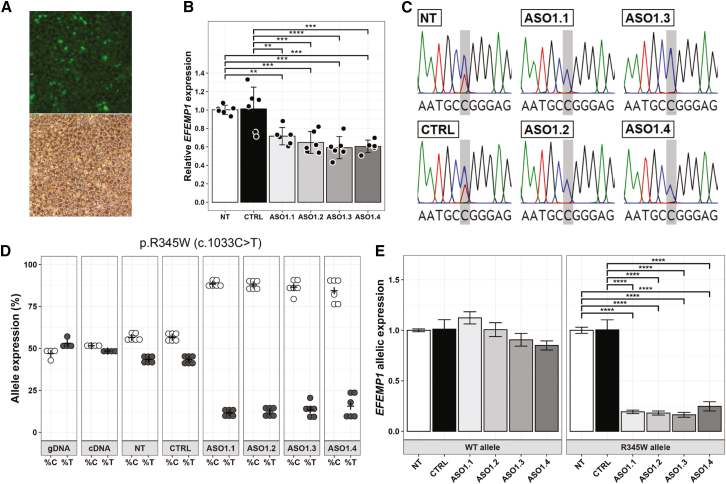
*EFEMP1* allele-specific targeting in iRPE following ASO transfection (A) Fluorescence and bright field images of 6-FAM conjugated ASO delivered to R345W iRPE. (B) Quantitation of *EFEMP1* transcript levels by qPCR in transfection reagent-treated (NT) R345W iRPE and following R345W iRPE transfection with ASO1.1, 1.2, 1.3, 1.4, or control ASO (CTRL) (200 nM) at day 72 of differentiation and qPCR analysis 48 h later. *N* = 3 independent experiments, *n* = 2 technical replicates of each condition per experiment. Statistical significance was determined by one-way ANOVA followed by post hoc Tukey’s (HSD) test, where ∗, ∗∗, and ∗∗∗ denote *p* values < 0.05, 0.01, and 0.005, respectively. ns, not significant. Bars represent mean ± SD. (C) Sanger sequencing chromatograms of transfection reagent-treated (NT) R345W iRPE and R345W iRPE treated with CTRL ASO, ASO1.1, ASO1.2, ASO1.3, or ASO1.4 (200 nM). (D) Quantitation of ASO-mediated allele-specific *EFEMP1* targeting by NGS. WT (%C) and R345W (%T) *EFEMP1* transcript levels were quantified in transfection reagent-treated R345W iRPE (NT) or R345W iRPE treated with CTRL or therapeutic ASO1.1, 1.2, 1.3, or 1.4. NGS results for gDNA and cDNA from the corresponding patient’s renal epithelial cells are also shown. *N* = 3 independent experiments, *n* = 2 technical replicates of each condition per experiment. Bars represent mean ± SD. (E) Integrated analysis of the qPCR and NGS data showing a significant reduction of the R345W transcript with all ASOs. Statistical significance was determined by one-way ANOVA followed by post hoc Tukey’s (HSD) test, where ∗, ∗∗, and ∗∗∗ denote *p* values < 0.05, 0.01, and 0.005, respectively. ns, not significant. Bars represent mean ± SD.

To assess the efficacy of ASO treatment in the downregulation of *EFEMP1* transcript expression, ASOs 1.1–1.4 were transiently transfected into R345W EFEMP1 patient-derived iRPE at a dose of 200 nM at day 72 of differentiation. Transfection of the 6-FAM-labeled ASO1.1 into the R345W iRPE resulted in strong intracellular fluorescence (Figure 4A). Experimental cell samples were lysed 48 h post-transfection for transcript-level analyses. qPCR analysis demonstrated a significant 30%–40% reduction in total *EFEMP1* transcript levels in therapeutic ASO-treated conditions, relative to transfection reagent only (NT) and control ASO-treated conditions (CTRL), suggesting an effective downregulation of *EFEMP1* expression by the designed ASOs (Figure 4B).

To explore allele-specificity, Sanger sequencing of cDNA amplicons spanning the c.1033C>T variation revealed preferential downregulation of the R345W transcript (Figure 4C). Electropherogram traces from therapeutic ASO-treated samples demonstrated marked resolution of the heterozygous “C/T” peak in the untreated (NT) and CTRL-treated R345W iRPE to a homozygous “C” peak in all ASO-treated samples, providing semi-quantitative confirmation of allele-specificity of the ASOs.

Targeted next-generation sequencing (NGS) was conducted using primers (Table S5), producing an amplicon covering the c.1033C>T locus of *EFEMP1* to enable quantitation of the allelic discrimination of the ASOs (Figure 4D). A baseline of allelic expression in the DHRD patient-derived samples was obtained to observe the possibility of *EFEMP1* allele skewing (Figure 4D). Amplicon quantitation of gDNA from patient-derived renal epithelial cells confirmed the expected 50:50 ratio between the WT and R345W alleles of *EFEMP1*, confirming equal copy number. cDNA prepared from renal epithelial cells of the patient also demonstrated a 50:50 ratio between the WT and R345W alleles of *EFEMP1*, indicating that in this cell type, both alleles of *EFEMP1* are equally expressed. However, basal expression levels of the cDNA prepared from differentiated iRPE in the NT and CTRL ASO-treated conditions revealed a 55:45 WT:R345W allelic expression split, indicating that the WT allele is expressed slightly higher than the R345W allele in differentiated iRPE. Following therapeutic ASO treatment, a clear reduction in R345W allele expression was observed for all ASOs, corroborating the Sanger sequencing data (Figure 4C). Therapeutic ASO treatment through transient transfection resulted in the WT transcript constituting 84.4%–88.3% of total allelic expression and the R345W transcript constituting 11.7%–15.6% of total allelic expression (Figure 4D).

Integrated analysis of the qPCR (Figure 4B) and NGS (Figure 4D) was performed to gain an understanding of how the ASO treatments affect the expression of the *EFEMP1* WT and R345W alleles relative to total *EFEMP1* transcript expression (Figure 4E). The percentage of allelic expression was calculated in comparison to total levels of *EFEMP1* transcript (Figure 4B) before normalization of the data to the NT control condition for the respective data group (WT or R345W allele) (Figure 4D). No significant differences between the control (NT and CTRL ASO) and therapeutic ASO treatment conditions were observed for the expression of the WT allele, with significant reductions in the R345W allele expression clearly observed following therapeutic ASO treatment. These results thus demonstrated consistent R345W allele-specific downregulation following treatment with the therapeutic ASOs. Based on these data, ASO1.1 was taken forward for the rest of the ASO treatments, due to its high reproducibility in reducing R345W *EFEMP1* allele expression and its ideal length (16-mer).

### Allele-specific *EFEMP1* targeting following ASO gymnosis of iRPE

**Figure 5:**
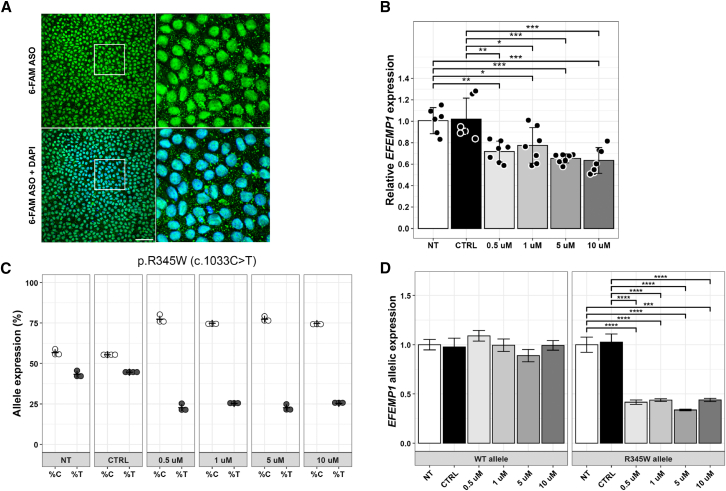
*EFEMP1* allele-specific targeting in iRPE following ASO gymnosis (A–C) R345W iRPE at day 60 of differentiation following a 7-day treatment with control ASO (CTRL) (1 μM) or ASO1.1 at the doses 0.5, 1, 5, and 10 μM. (A) Fluorescence from gymnotically delivered 6-FAM-conjugated ASO1.1 (1 μM). The white rectangle (inset) demarcates the magnified image shown on the right. Nuclei are labeled with DAPI. Scale bars, 50 μm. (B) Quantitation of *EFEMP1* transcript levels by qPCR in untreated (NT) R345W iRPE, CTRL ASO-treated R345W iRPE, and R345W iRPE treated with increasing doses of ASO1.1 (0.5, 1, 5, and 10 μM). *N* = 3 independent experiments. Statistical significance was determined by one-way ANOVA followed by post hoc Tukey’s (HSD) test, where ∗, ∗∗, and ∗∗∗ denote *p* values < 0.05, 0.01, and 0.005, respectively. Bars = mean ± SD. (C) Quantitation of ASO1.1-mediated allele-specific *EFEMP1* targeting by NGS at doses 0.5, 1, 5, and 10 μM. WT (%C) and R345W (%T) *EFEMP1* transcript levels were quantified in untreated R345W iRPE (NT) or R345W iRPE treated with CTRL or increasing doses of ASO1.1 (0.5, 1.0, 5.0, and 10 μM). *N* = 3 independent experiments. (D) Integrated analysis of the qPCR and NGS data showing a significant reduction of the R345W transcript with all ASO1.1 doses. Statistical significance was determined by one-way ANOVA followed by post hoc Tukey’s (HSD) test, where ∗, ∗∗, and ∗∗∗ denote *p* values < 0.05, 0.01, and 0.005, respectively. Bars = mean ± SD.

Gymnotic (unassisted) delivery of ASO1.1 at day 60 of differentiation was carried out to evaluate ASO efficacy in a setting that more closely recapitulates physiological uptake mechanisms (Figure 5). Fluorescence microscopy of gymnotically delivered 6-FAM-conjugated ASO1.1 (1 μM) showed efficient uptake across the R345W iRPE population following a 7-day treatment (Figure 5A). The ASO was demonstrated to robustly accumulate in the nucleus, with additional punctate localization in the cytoplasm. This distribution is consistent with the known subcellular activity of RNase H1, which is present in both the nucleus and cytoplasm.^22^ While the ASO is designed to target mature mRNA, its nuclear localization suggests potential additional engagement with pre-mRNA targets. RNase H-mediated cleavage of the target transcript can occur in both subcellular compartments, with cytoplasmic ASO signal potentially indicating interactions with mature transcripts or transient endosomal retention. Thus, ASO localization in both of these compartments indicates enhanced likelihood of effective target knockdown following gymnotic uptake.

R345W iRPE underwent a 7-day treatment with ASO1.1 at the doses 0.5, 1, 5, and 10 μM, and *EFEMP1* gene expression was analyzed by qPCR (Figure 5B). Therapeutic ASO treatments at each of the doses led to a significant downregulation of total *EFEMP1* expression relative to the NT and CTRL ASO treatment conditions. However, no clear dose-dependent response was observed between therapeutic ASO treatments. The magnitude of *EFEMP1* knockdown remained comparable between the lowest and highest ASO doses tested. This plateau in response indicates that maximal target suppression could be achieved at or below the lowest tested concentration.

Next, targeted NGS was employed to evaluate the allelic discrimination of gymnotically delivered ASOs (Figure 5C). Baseline allelic expression in iRPE for the DHRD patient included in this study was confirmed at a 55:45 WT:R345W allelic split, as observed in the NT and CTRL ASO conditions. Again, there was no dose-dependent response following ASO1.1 treatment at 0.5, 1, 5, and 10 μM, as all doses led to the WT transcript constituting 73%–78% and the mutant transcript constituting 22%–27% of total *EFEMP1* expression.

Integrated analysis of the qPCR (Figure 5B) and targeted NGS (Figure 5C) data was carried out to explore the efficacy of ASO1.1 in its allele-specific downregulation of *EFEMP1*. The percentage of allelic expression was calculated in comparison to total levels of *EFEMP1* transcript (Figure 5B) with normalization of the data to the NT control condition for the respective data group (WT or R345W allele) (Figure 5D). Similar to the qPCR and NGS data collation carried out for the transient transfection (Figure 4D), no significant differences between the controls (NT and CTRL ASO) and therapeutic ASO treatment conditions were observed for WT allele expression, whereas significant reductions in mutant *EFEMP1* were clearly observed. Overall, this confirmed consistent *EFEMP1* R345W allele-specific downregulation via ASO1.1 gymnosis.

### Rescue of APOE and lipid accumulation in patient iRPE following ASO gymnosis

**Figure 6:**
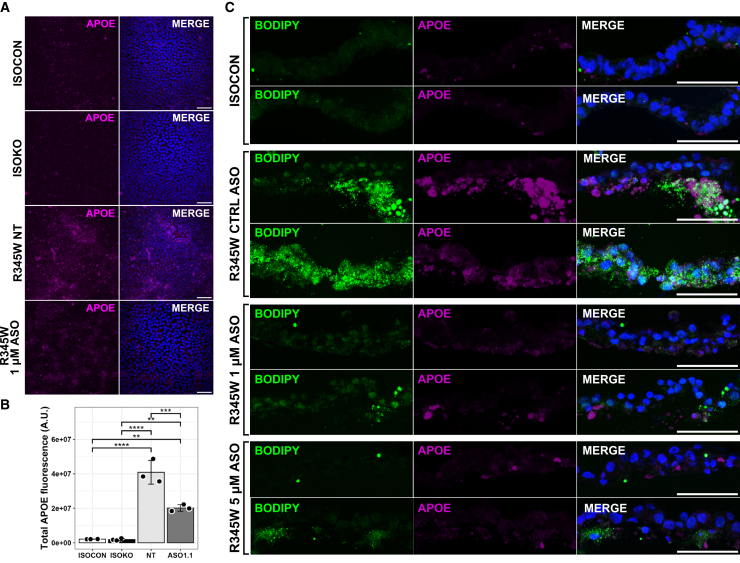
Phenotypic rescue of APOE and lipid accumulation in R345W iRPE following ASO gymnosis (A) IF of APOE (magenta) in ISOCON, ISOKO, untreated R345W (NT), and R345W iRPE treated gymnotically with ASO1.1 (1 μM dose for 7 days from day 60 of differentiation). APOE IF of R345W iRPE treated with ASO (1 μM) was acquired from the same confocal field shown in Figure 5A to confirm successful ASO delivery, with 6-FAM ASO and APOE IF presented as separate channels. Nuclei are labeled with DAPI. Scale bars, 50 μm. (B) Quantitation of APOE (relative fluorescence levels) in ISOCON, untreated R345W iRPE (NT), and ASO1.1 treated R345W iRPE (1 μM dose for 7 days from day 60 of differentiation). Fluorescence levels were quantified from three independent images, AU, arbitrary units. Statistical significance was determined by one-way ANOVA followed by post hoc Tukey’s (HSD) test, where ∗, ∗∗, and ∗∗∗ denote *p* values < 0.05, 0.01, and 0.005, respectively. Bars = mean ± SD. (C) Images of transverse sections (14 μM) from ISOCON and R345W iRPE treated gymnotically with CTRL ASO or with therapeutic ASO1.1 (1 and 5 μM doses for 14 days from day 84 of differentiation). Images show neutral lipid (BODIPY, green) and basal APOE (magenta). Nuclei are labeled with DAPI. Scale bars, 20 μm.

We next examined the phenotype of patient iRPE following gymnotic treatment with the therapeutic ASO1.1 (Figure 6). To assess the impact of ASO gymnosis on APOE, iRPE samples following a 1-week treatment starting from day 60 of differentiation were immunolabelled for APOE (Figure 6A). APOE has been shown to accumulate in sub-RPE deposits and contribute to drusen formation, hallmarks of DHRD. IF analysis revealed a low APOE signal for both the ISOCON and ISOKO iRPE. In contrast, NT R345W iRPE exhibited a marked increase in APOE fluorescence, consistent with the DHRD disease phenotype. 1-week gymnosis of ASO1.1 at a dose of 1 μM resulted in a substantial reduction in APOE fluorescence, as illustrated in the representative images and by quantification of total fluorescence intensity (Figures 6A and 6B). Quantitative analysis confirmed a significant elevation of APOE levels in the R345W NT condition relative to ISOCON and ISOKO iRPE. There was no difference in the levels of APOE quantified in the ISOCON and ISOKO iRPE. APOE levels in the ASO-treated R345W iRPE displayed significantly reduced APOE fluorescence relative to the NT condition, although expression remained modestly higher than in the ISOCON and ISOKO conditions, following the 1-week treatment. These findings indicate that suppression of R345W *EFEMP1* via gymnotically delivered ASO mitigates downstream APOE accumulation, implicating a functional relationship between *EFEMP1*-driven pathology and APOE accumulation in this iRPE model.

Next, the distribution of APOE and lipid accumulation in vertically embedded iRPE cross sections was assessed following a 2-week gymnotic ASO1.1 treatment, initiated on day 84 of differentiation, at concentrations of 1 and 5 μM (Figure 6C). Sections were stained with BODIPY to visualize neutral lipid content and co-labeled for APOE. ISOCON iRPE exhibited minimal BODIPY and APOE immunoreactivity, whereas patient-derived iRPE treated with CTRL ASO displayed elevated fluorescence signals for both markers. In contrast, ASO1.1-treated iRPE showed a pronounced reduction in BODIPY and APOE fluorescence intensity at both 1 and 5 μM doses, indicating that gymnotic ASO1.1 administration effectively decreases APOE expression and lipid accumulation in patient-derived DHRD iRPE within 2 weeks.

**Figure 7:**
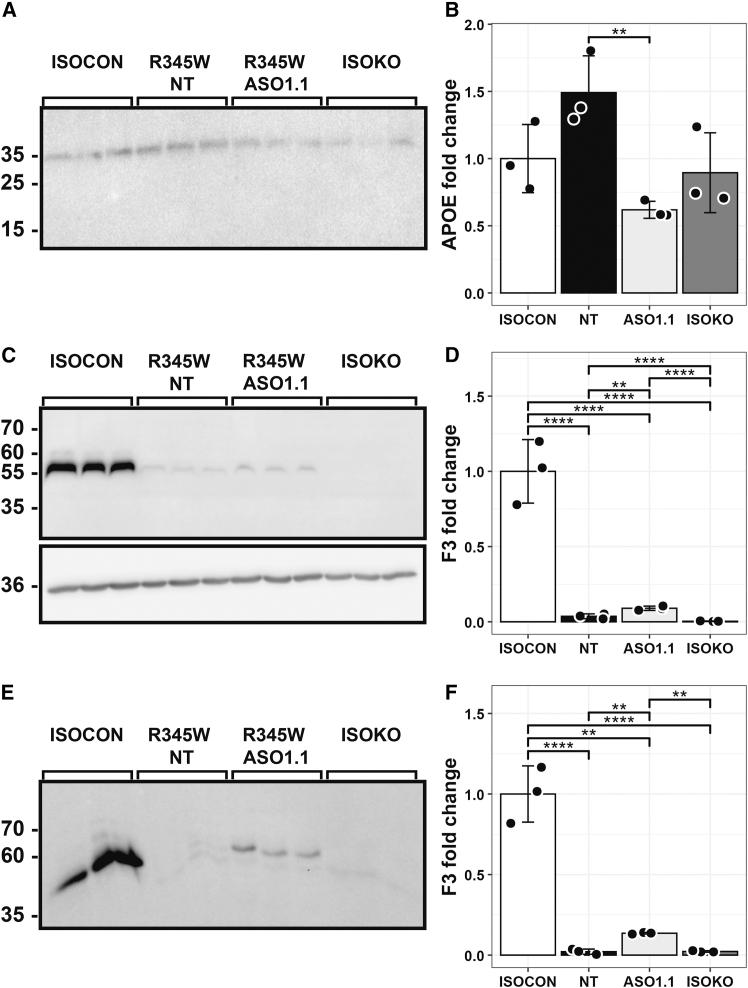
APOE and EFEMP1 protein levels in R345W iRPE following ASO gymnosis (A) Western blot analysis of APOE (∼35 kDa) in SDS-solubilized pellet fractions from ISOCON, NT R345W, ASO1.1 treated R345W, and ISOKO iRPE. (B) ImageJ densitometric quantitation of APOE levels from ISOCON, NT R345W, ASO1.1 treated R345W, and ISOKO iRPE normalized to total protein levels (Ponceau S). *N* = 3 independent protein isolates per condition. Statistical significance was determined by two-way unpaired Student’s *t* test where ∗, ∗∗, ∗∗∗, and ∗∗∗ denote *p* values < 0.05, 0.01, 0.001, and 0.0001, respectively. Bars = mean ± SD. (C) Western blot analysis of EFEMP1 (F3) (∼55 kDa) (upper) and GAPDH (∼36 kDa) (lower) in cell lysates from ISOCON, NT R345W, ASO1.1 treated R345W and ISOKO iRPE. (D) ImageJ densitometric quantitation of EFEMP1 (F3) levels in soluble cell lysates from ISOCON, NT R345W, ASO1.1 treated R345W, and ISOKO iRPE, normalized to GAPDH. *N* = 3 independent protein isolates per condition. Statistical significance was determined by two-way unpaired Student’s *t* test where ∗, ∗∗, ∗∗∗, and ∗∗∗ denote *p* values < 0.05, 0.01, 0.001, and 0.0001, respectively. Bars = mean ± SD. (E) Western blot analysis of EFEMP1 (F3) in SDS-solubilized pellet fractions from ISOCON, NT R345W, ASO1.1-treated R345W, and ISOKO iRPE. (F) ImageJ densitometric quantitation of EFEMP1 (F3) levels in SDS-solubilized pellet fractions from ISOCON, NT R345W, ASO1.1-treated R345W, and ISOKO iRPE, normalized to Ponceau S total protein levels. *N* = 3 independent protein isolates per condition. Statistical significance was determined by two-way unpaired Student’s *t* test where ∗, ∗∗, ∗∗∗, and ∗∗∗ denote *p* values < 0.05, 0.01, 0.001, and 0.0001, respectively. Bars = mean ± SD.

To confirm these findings, we conducted western blot analysis following the 2-week gymnotic ASO1.1 treatment (1 μM). APOE resolved to the expected molecular weight of ∼35 kDa and was detected in the SDS soluble cell pellet (Figure 7A) in accordance with the sub-RPE basal localization. Elevated levels of APOE were detected in NT R345W iRPE in comparison to ISOCON and ISOKO iRPE, and these levels were reduced in ASO1.1-treated R345W iRPE (Figure 7A). Densitometric quantification, normalized to total protein levels in the insoluble fraction, confirmed an increase in APOE levels in NT R345W iRPE that were significantly decreased following ASO1.1 treatment (Figure 7B). These results confirm that allele-specific ASO-mediated knockdown of the *EFEMP1* R345W transcript alleviates APOE accumulation in the iRPE model.

We next investigated EFEMP1 (F3) levels in the iRPE model following the 2-week ASO1.1 treatment (1 μM) (Figures 7C, 7D, 7E, and 7F) by western blot analysis. In soluble cell lysates and SDS-solubilized cell pellets, EFEMP1 resolved to the expected molecular weight of ∼55 kDa (Figures 7C and E). In soluble cell lysates, the highest levels of EFEMP1 were detected in ISOCON iRPE with significantly reduced levels detected in NT R345W iRPE and no EFEMP1 detected in ISOKO iRPE as expected, confirming the antibody specificity (Figure 7C, upper). An increase in EFEMP1 levels was observed in ASO1.1 (1 μM) treated R345W iRPE, although these levels did not reach those in the ISOCON iRPE (Figure 7C, upper). These results were confirmed by densitometric quantification of EFEMP1 levels (Figure 7D) normalized to GAPDH (Figure 7C, lower). Similarly, in the SDS solubilized pellet fraction, the highest levels of EFEMP1 were detected in ISOCON iRPE with no EFEMP1 detected in ISOKO iRPE (Figure 7E). EFEMP1 was almost undetectable in NT R345W iRPE but the rescue of EFEMP1 levels in this fraction was once again observed following ASO1.1 (1 μM) treatment (Figure 7E). Densitometric quantification of the EFEMP1 levels in the insoluble fraction, normalized to total protein levels, confirmed these findings with a significant increase in EFEMP1 levels following ASO1.1 treatment (Figure 7F). Although the EFEMP1 signal was detected in the ASO-treated R345W iPSC, the relative contribution of WT versus R345W protein to this band cannot be determined in the absence of protein variant-specific approaches. Based on these observations, we hypothesize that the data suggest that the R345W has a dominant negative effect on WT EFEMP1 that renders it undetectable by western blot analysis in the cell lysate and pellet fractions, potentially related to solubility and/or epitope availability, in accordance with the reported altered structure, increased stability, and accumulation of R345W EFEMP1 in the ECM.^5^^,^^6^^,^^7^^,^^8^

Finally, the consequences of lipid and APOE accumulation on the ability of iRPE to phagocytose photoreceptor outer segments (POS) were investigated. Under the conditions tested, we did not detect a significant difference in the ability of the ISOCON, NT R345W, and ASO1.1-treated R345W iRPE to phagocytose POS (Figure S3).

### Rescue of patient iRPE phenotype following long-term ASO gymnosis

**Figure 8:**
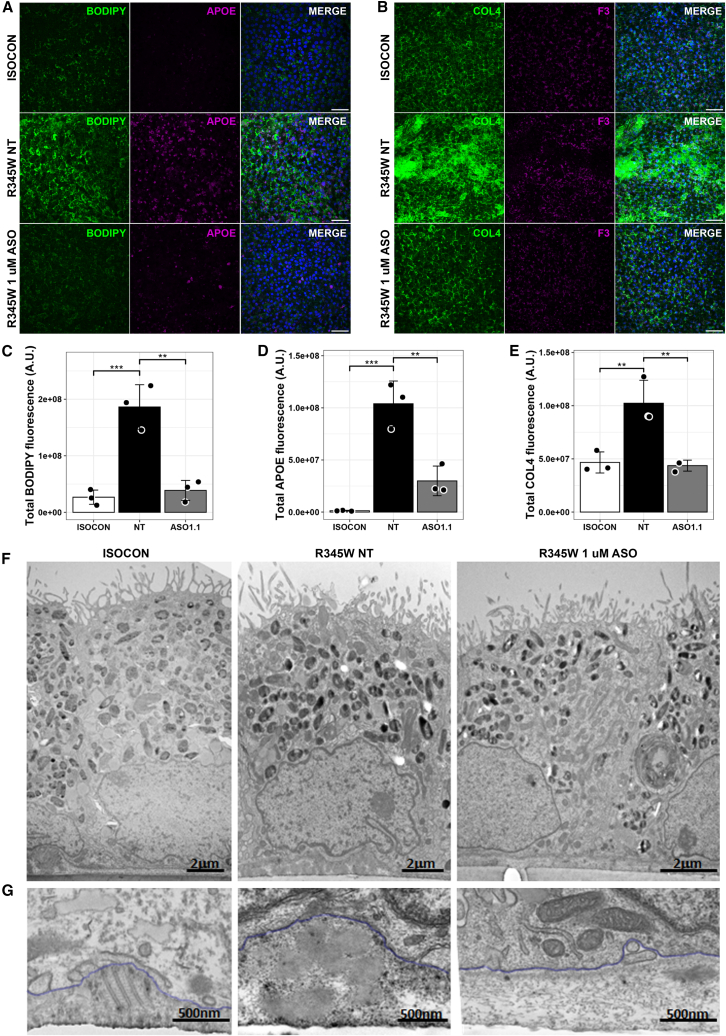
Phenotypic rescue of APOE, ECM abnormalities, and sub-RPE lipid deposition in R345W iRPE following ASO gymnosis (A) Staining of neutral lipids (BODIPY, green) and APOE (magenta) in ISOCON, untreated R345W (NT), and R345W iRPE following 2-month ASO1.1 gymnosis (1 μM) from day 100 of differentiation. Nuclei are labeled with DAPI. Scale bars, 50 μm. (B) Staining of COL4 (green) and F3 (magenta) in ISOCON, untreated R345W (NT), and R345W iRPE following 2-month ASO1.1 gymnosis (1 μM) from day 100 of differentiation. Nuclei are labeled with DAPI. Scale bars, 50 μm. (C–E) Quantitation of relative fluorescence levels of BODIPY (C), APOE (D), and COL4 (E). Fluorescence levels were quantified from three independent images, AU, arbitrary units. Statistical significance was determined by one-way ANOVA followed by post hoc Tukey’s (HSD) test, where ∗, ∗∗, and ∗∗∗ denote *p* values < 0.05, 0.01, and 0.005, respectively. Bars = mean ± SD. (F) TEM of ISOCON, untreated R345W (NT), and R345W iRPE following 2-month ASO1.1 gymnosis (1 μM) from day 100 of differentiation. TEM of ISOCON and untreated R345W (NT) as in Figure 2D (upper). Upper: ISOCON, untreated R345W, and ASO1.1 treated R345W iRPE. Scale bars, 2 μm. Lower: representative TEM images of basal deposits between the basal membrane (blue outline) and the underlying transwell membrane. Scale bars, 500 nm.

To further investigate the robustness of ASO1.1 effects and its potential impact on ECM organization, a 2-month gymnotic treatment was conducted on patient-derived iRPE (Figure 8). ISOCON, NT R345W, and 1 μM ASO-treated R345W iRPE cultured on chamber slides were analyzed for BODIPY, APOE, COL4, and F3 expression and localization. Consistent with our earlier findings, ISCOCON iRPE shows minimal BODIPY and APOE signal (Figure 8A), indicating low lipid and APOE accumulation. Conversely, the R345W NT condition exhibited strong immunostaining for both markers. ASO1.1 treatment (1 μM) in R345W iRPE significantly reduced both BODIPY and APOE fluorescence (Figures 8A–8C and 8D). IF was also conducted with antibodies to COL4 and F3 (Figure 8B). ISOCON iRPE showed a homogenous distribution of COL4 staining, in contrast to uneven patches of accumulated COL4 observed in the NT R345W condition (Figure 8B). ASO1.1 treatment significantly attenuated COL4 accumulation, resulting in a more even staining pattern (Figures 8B and E) and supporting a potential role for mitigating ECM disorganization induced by R345W *EFEMP1* expression. These findings indicate that the reduction of R345W *EFEMP1* expression not only attenuates lipid and APOE accumulation but also promotes the rescue of pathological ECM features in DHRD iRPE.

Finally, TEM of ISOCON, NT R345W, and ASO1.1-treated R345W iRPE was conducted at day 158 following 2-month ASO1.1 gymnosis (1 μM) commencing at day 100 of differentiation. All cells showed characteristic RPE-like morphology, forming monolayers of polarized pigmented cells (Figure 8F). Accumulation of microfibrils (blue outline) was observed in the ISOCON iRPE between the basal membrane and the underlying transwell membrane (24% of surface) (Figure 8G). In contrast, lipid-rich deposits (blue outline) were observed in the NT R345W-iPSC (22% of surface) with microfibril accumulation in the ECM observed only rarely (5% of surface) (Figure 8G). Lipid-rich deposits were almost completely absent (3% of surface) from R345W iRPE following 2-month gymnosis with ASO1.1 (1 μM) (Figure 8G).

## Discussion

In this study, we confirm that both patient-derived and isogenic iRPE initially exhibit a typical RPE-like morphology and express key RPE markers associated with epithelial identity and polarity, tight junction formation, and melanosome biogenesis. However, the c.1033C>T, p.(Arg345Trp) variant results in progressive phenotypic alterations in the patient-derived iRPE. While the corrected isogenic iRPE formed a well-organized, uniform monolayer, the patient-derived iRPE fails to form a uniform monolayer and becomes progressively less well-organized, with tight junctions appearing more fragmented and discontinuous. Moreover, the basement membrane in the isogenic control iRPE is well-organized, forming a continuous and uniform basal layer. In contrast, a dysmorphic basal layer characterized by progressive disruption and structural disorganization of the ECM and thickening of the basement membrane is evident in the patient-derived iRPE, indicating abnormalities in the structural integrity of this layer. Interestingly, EFEMP1 itself is localized across the basement membrane in both isogenic and patient-derived iRPE. However, in patient-derived iRPE, EFEMP1 accumulated in the basement membrane and presented a more filamentous appearance. This is consistent with the accumulation of EFEMP1 in the basal layer between RPE and drusen in human DHRD donor eyes, and with the accumulation of Efemp1 in basal deposits in primary mouse RPE cells carrying the R345W *Efemp1* substitution and in sub-RPE deposits in *Efemp1* knockin mice.^7^^,^^8^^,^^11^^,^^23^ Finally, the accumulation of lipids and APOE, a key component of drusen, is evident in the patient-derived iRPE. While lipid-rich deposits underneath the RPE in the basal layer were seldom observed in the isogenic iRPE, increased sub-RPE lipid-rich deposits were observed in the patient-derived iRPE. Similar phenotypic characteristics have been reported in other models of DHRD using patient-derived iRPE and isogenic controls.^13^^,^^14^ Galloway et al. reported an increased accumulation of lipids and APOE-positive basal deposits beneath the RPE in patient-derived iRPE, in addition to increased deposition and levels of ECM and basement membrane constituents.^13^ Similarly, Tsai et al. reported the intracellular and extracellular accumulation of lipids in patient-derived iRPE, in addition to the downregulation of carboxylesterase 1 (CES1) and the attenuation of cholesterol efflux from these cells.^14^ The authors proposed that a hyper-inhibitory effect of R345W EFEMP1 on EGFR signaling may suppress CES1 expression, thereby attenuating cholesterol export and leading to lipid accumulation.

In addition to the phenotypic alterations described above, altered mitochondrial morphology is a novel phenotype observed in the patient-derived iRPE, including a population of elongated mitochondria and, for some mitochondria, a longitudinal orientation of the cristae. Mitochondrial dysfunction, together with oxidative stress and inflammation, is increased in age-related macular degeneration (AMD)^24^^,^^25^^,^^26^ but has not been previously reported in juvenile macular dystrophy, raising the possibility of a direct role for mitochondrial dysfunction in the disease pathology of these disorders. Another novel finding in our study was the occurrence of microfibrils in the ECM beneath the basement membrane in the isogenic iRPE, which was largely absent from the patient-derived iRPE. The ultrastructural appearance and banded pattern of these microfibrils in the ECM suggest that they may be composed of collagen VI.^27^ While the identity of these microfibrils in the ECM requires further scrutiny, it has been reported that ARPE-19 cells engineered to be homozygous for the R345W *EFEMP1* variant developed changes in the ECM that included a reduced and disorganized network of stretched collagen VI fibers in comparison to WT ARPE-19, which exhibited a continuous network of fibers with some thickened and localized areas.^28^ This was accompanied by a thickened and multilayered fibronectin network in the R345W ARPE-19 cells.^28^ Overall, the data are consistent with structural disorganization of the ECM in the cellular models of DHRD.

Importantly, the isogenic *EFEMP1* knockout iRPE in our study did not emulate the phenotypic characteristics observed in the patient-derived iRPE. The *EFEMP1* knockout iRPE formed a well-organized monolayer with a continuous and uniform basement membrane, indicating maintenance of the structural integrity of the ECM. Moreover, APOE accumulation was not observed in the *EFEMP1* knockout iRPE. Although this finding cannot fully rule out subtle or undetected changes in the RPE basal layer and ECM, it does confirm the dominant toxic gain-of-function of R345W EFEMP1 in the RPE in addition to the absence of *EFEMP1* loss of function or haploinsufficiency as a disease mechanism underpinning DHRD pathology.

Given the toxic gain of function of R345W EFEMP1, we designed an antisense oligonucleotide to specifically promote the downregulation of the R345W *EFEMP1* transcript through RNase H-dependent degradation. Both assisted delivery of the lead therapeutic ASO at a dose of 200 nM and gymnotic delivery over a dose range of 0.5–10 μM resulted in allele-specific targeting of the R345W *EFEMP1* transcript in the patient-derived iRPE. ASO treatment of patient-derived iRPE through transient transfection resulted in the reduction of R345W *EFEMP1* transcript levels to 11.7%–15.6% of total allelic expression as quantified by NGS, while gymnotic treatment was less effective, reducing R345W *EFEMP1* transcript levels to 22%–27% of total levels. A dose-dependent response was not observed following gymnotic delivery of the lead ASO, with the magnitude of knockdown of the R345W *EFEMP1* transcript comparable between the lowest and highest ASO doses tested, indicating that maximal target suppression could be achieved at or below the lowest tested concentration. Despite the reduced efficacy of the lead ASO to reduce the R345W *EFEMP1* transcript following gymnotic delivery, treatment of patient-derived iRPE with the lead ASO was able to effectively rescue the disease phenotype even when treatment was initiated after the onset of the phenotypic changes and within a short treatment time frame. A 1-week gymnotic treatment with ASO resulted in a substantial reduction in drusen-associated APOE accumulation. Similarly, a 2-week gymnotic ASO treatment administered after progression of the phenotypic changes in the patient-derived iRPE resulted in a pronounced reduction not only of APOE but also of lipid accumulation. Interestingly, while a significant accumulation of EFEMP1 was evident in the ECM beneath the RPE in patient-derived iRPE, the c.1033C>T, p.(Arg345Trp) variation rendered EFEMP1 virtually undetectable by western analysis in comparison to the isogenic control iRPE. The EFEMP1 antibody used in this study was raised against amino acids 107–493; however, the precise epitope recognized is not defined, and the specificity of the antibody for WT or R345W EFEMP1 is not known. Therefore, it is unclear whether the R345W substitution affects antibody binding, and differences in epitope accessibility between WT and R345W EFEMP1 cannot be excluded. Based on these observations, we hypothesize that the accumulation of R345W EFEMP1 in the ECM exerts a dominant negative effect on WT EFEMP1, either rendering the protein insoluble within the highly cross-linked and disorganized ECM or as a result of a change in the structural integrity and therefore epitope availability. ASO treatment partially restored EFEMP1 immunoreactivity; however, the extent to which this reflects selective reduction of R345W and/or improved detectability of WT protein cannot be determined from the current data. These findings are therefore consistent with, but do not directly demonstrate, a relief of the proposed dominant-negative effect. Finally, a 2-month gymnotic treatment in aged patient-derived iRPE was shown to not only attenuate APOE and sub-RPE lipid deposits but also rescue the accumulation of collagen IV and disorganization of the ECM. Overall, the results suggest that administration of ASO treatment after the onset of drusen formation in DHRD patients could be therapeutically beneficial in ameliorating the pathological features of the disease.

Currently, the clinical care of DHRD patients includes the use of low-vision aides to assist with daily living and regular observation and retinal imaging to monitor drusen and detect complications such as choroidal neovascularization (CNV). Interventions targeting complications that occur in some DHRD patients, such as CNV, include anti-vascular endothelial growth factor (VEGF) intravitreal injections (bevacizumab or ranibizumab). Photodynamic laser therapy to treat CNV and low-energy subthreshold nanosecond laser (2RT) treatment have also been trialed in a small number of DHRD patients.^29^^,^^30^ In comparison, our antisense oligonucleotide is the first to target the cause of DHRD at the source and could potentially be used to treat all DHRD patients. Although DHRD is rare (*EFEMP1* c.1033C>T allele frequency is 1.23 × 10^−5^ in gnomAD database v4.1.0), it is a monogenic, single variant disorder with all DHRD patients worldwide harboring the *EFEMP1* c.1033C>T, p.(Arg345Trp) variant. In an investigation of the spectrum of genetic variants in the most common genes causing inherited retinal disease in a large molecularly characterized cohort in the United Kingdom, the *EFEMP1* c.1033C>T, p.(Arg345Trp) variant was identified as one of the top twenty most prevalent variants identified.^31^ Progression of the ASO developed in this study toward clinical translation will require further pre-clinical development, including *in vivo* safety profiling and pharmacology. Notably, while two *Efemp1* R345W knockin models have been developed, there is a 3 bp mismatch in the ASO target sequence between mouse *Efemp1* (GenBank: NM_146015.2) and human *EFEMP1* (GenBank: NM_001039348.3), making the mouse models unsuitable to test the *in vivo* efficacy of the human-specific ASO1.1.^8^^,^^23^ Consequently, *in vivo* studies performed in WT mice would assess general tolerability of the ASO chemistry rather than on-target activity or target-specific safety. In addition, comprehensive biodistribution and pharmacokinetic profiling of ASO1.1 remain to be established and represent important next steps for clinical translation. Interestingly, the intravitreally delivered ASO Sepofarsen, a splice-modulating ASO targeting the deep intronic variant *CEP290* c.2991 + 1655A>G, causing Leber congenital amaurosis type 10 (LCA10), entered clinical trials in the absence of a suitable animal disease model,^32^^,^^33^ thereby setting a precedent for the rapid progression of therapeutic nucleic acids into clinical trials in the absence of appropriate animal models.

In conclusion, the allele-specific antisense oligonucleotide developed in this study could be a viable therapeutic option for DHRD patients, with the potential to alleviate the underlying pathology, including sub-RPE deposition of drusen-associated components and structural reorganization of the RPE ECM, ultimately improving clinical outcomes.

## Materials and methods

### EFEMP1 plasmids

*EFEMP1* (ENST00000355426.8) was amplified from cDNA prepared from retinal organoids as described in Sai et al.^34^ USER cloning technology (NEB, Hitchin, UK) was used according to the manufacturer’s instructions to insert the *EFEMP1* open reading frame, excluding the stop codon into the mammalian expression vector p3xFLAG-CMV-14 (E7908) (Sigma-Aldrich, Gillingham, UK) and pmScarlet_C1 (Addgene, plasmid #85042), resulting in in-frame fusion of EFEMP1 with a C-terminal 3×FLAG tag (pEFEMP1-3× FLAG) and C-terminal mScarlet tag (pEFEMP1-mScarlet), respectively. The *EFEMP1* c.1033C>T, p.(Arg345Trp) variation and a Kozak sequence upstream of the *EFEMP1* open reading frame were introduced into pEFEMP1-mScarlet by site-directed mutagenesis using the Q5 site-directed mutagenesis kit (NEB) to generate pEFEMP1(R345W)-mScarlet. The primer sequences for *EFEMP1* amplification and sequencing are in Table S5.

### ASO screening in HEK293T cells

HEK293 cells (ATCC) were cultured with DMEM (Life Technologies, Waltham, MA, USA) with 10% fetal bovine serum (Thermo Fisher Scientific, Paisley, UK) at 37°C with 5% CO_2_. HEK293T cells were seeded in 24-well plates at a density of 0.7 × 10^5^ cells per well and transfected 24 h later using lipofectamine 2000 (Thermo Fisher Scientific), as described by the manufacturer. HEK293T cells were transfected with both pEFEMP1(WT)-3×FLAG (800 ng) and pEFEMP1(R345W)-mScarlet (800 ng), and with control ASO (CTRL) (50 nM) or increasing concentrations (25, 50, 100, and 200 nM) of therapeutic ASO. Results were compared to untreated cells transfected with both plasmids (plasmids only). The RNA was extracted 48 h post-treatment with the RNeasy Mini kit (Qiagen, Manchester, UK) and cDNA synthesized from RNA samples using the Tetro cDNA Synthesis kit (Bioline, Meridian Bioscience Europe, London, UK), according to the manufacturer’s protocol. qPCR was conducted using primer pairs specific for WT *EFEMP1*, R345W *EFEMP1*, or total *EFEMP1* to quantify the levels of *EFEMP1* transcript using an Applied Biosystems QuantStudio 6 Flex real-time PCR system and the ΔΔCt method. Gene expression was normalized to the housekeeping genes *GAPDH* and *ACTB*, and all values were expressed relative to the untreated ‘plasmid only’ condition. Primer sequences for *EFEMP1* qPCR are in Table S5.

### Urine collection and renal epithelial cell isolation and expansion

Urine was collected from a DHRD patient harboring the c.1033C>T, p.(Arg345Trp) variant in *EFEMP1*. Collection, isolation, and expansion of renal epithelial cells was carried out similarly to protocols described by Zhou et al. and Hildebrand et al.^35^^,^^36^ Briefly, the urine sample (around 50 mL) was centrifuged at 400 × *g* for 10 min. The supernatant was discarded, and the pellet was washed with 10 mL of Dulbecco’s phosphate-buffered saline (Thermo Fisher Scientific) containing 1× Anti-Anti (Thermo Fisher Scientific) and centrifuged at 400 × g for 10 min. The pellet was resuspended in 2 mL primary medium, consisting of DMEM/Ham’s F-12 nutrient mix (1:1) (Thermo Fisher Scientific), with 10% of fetal bovine serum (FBS; Thermo Fisher Scientific), renal cell growth medium (REGM) SingleQuot kit supplements (Lonza, Cambridge, UK), and 1× Anti-Anti. The cells were seeded into a single well of a 12-well plate coated with 0.1% gelatin. One mL of primary medium was added to the wells at 24, 48, and 72 h following plating, without removal of any media. Renal epithelial cell colonies appeared within 3 days. At 96 h post-seeding, most of the medium was aspirated and replaced by proliferation medium, consisting of a 1:1 mixture of renal epithelial cell growth basal medium (REBM) supplemented with REGM SingleQuots (Lonza) and DMEM high glucose (Thermo Fisher Scientific) supplemented with 10% FBS, 1% GlutaMAX, 1% non-essential amino acids (NEAA), and 1× Anti-Anti. Half media changes were performed every day. Fourteen days following urine collection, cells reached 90% density and were dissociated using TrypLE Express (Thermo Fisher Scientific) for 10 min at 37°C, before being seeded at a ratio of 1:4 onto gelatin-coated wells. Cells were expanded for a maximum of four passages.

### Human iPSC reprogramming and culture

Renal epithelial cells passaged less than 3 times were used to generate iPSCs. Cells (5 × 10^5^) were dissociated using TrypLE Express (Thermo Fisher Scientific) for 10 min at 37°C and electroporated with four integration-free episomal vectors—pCXLE-hOCT3/4-shp53-F (Addgene, plasmid #27077), pCXLEhSK (Addgene, plasmid #27078) containing SOX2 and KLF4, pCXLEhUL (Addgene, plasmid #27080) containing L-MYC and LIN28, and miRNA 302/367 plasmid (Gift from Dr. J.A. Thomson, Regenerative Biology, Morgridge Institute for Research, Madison, Wisconsin, USA; Howden et al.)—using the Amaxa Basic Nucleofector kit for primary mammalian epithelial cells, program T-020 (Lonza). Electroporated cells were seeded onto 1% Geltrex-coated (Thermo Fisher Scientific) plates and cultured in StemFlex media (Thermo Fisher Scientific). Media changes were carried out every other day. iPSC colonies were picked around day 14, expanded in Stem Flex media on Geltrex-coated 6-well plates, and routinely passaged using cell dissociation buffer (Thermo Fisher Scientific). iPSC line pluripotency was confirmed using iPSC-specific antibodies: anti-OCT4 (Abcam, Cambridge, UK), anti-Nanog (Abcam), anti-SSEA4 (Cell Signaling Technologies, Cambridge, UK), and anti-TRA-1-80 (Thermo Fisher Scientific) (Table S6).

### Generation of isogenic iPSC lines

To generate an isogenic control line (ISOCON), a 20 nt gRNA (NGG PAM) and 127 nt ssODN template were designed for the correction of the *EFEMP1* c.1033C>T allele through CRISPR-mediated HDR. The ssODN repair template incorporated the desired correction and a synonymous PAM change to prevent further Cas9-mediated cleavage of the edited DNA. To generate an *EFEMP1* knockout (ISOKO) iPSC line, CRISPR-mediated NHEJ in the patient iPSC line was employed. A 20 nt gRNA with a canonical PAM (NGG) was designed to target exon 5 of *EFEMP1* (Table S2). The crRNA and ssODN (with phosphorothioate [PS] modifications to the ends) were obtained from Integrated DNA Technologies (IDT, Bristol, UK). Sequences for crRNAs and ssODNs are in Tables S1 and S2. tracrRNA, crRNA, and ssDNA donors were resuspended in nuclease-free ddH_2_O to 100 μM. The crRNA and tracrRNA were combined and heated at 95°C for 5 min to form a 50 μM gRNA complex. Three μL of gRNA complexes were then added to 2 μL of 61 μM Alt-R Cas9 enzyme (IDT) to generate ribonucleoprotein (RNP) complexes and incubated at room temperature (RT) for 20 min. iPSCs were cultured in Stemflex (Gibco, Thermo Fisher Scientific) with 10 μM ROCK inhibitor Y-27632 (STEMCell Technologies, Cambridge, UK) for 2 h prior to single-cell dissociation with TrypLE (Gibco, Thermo Fisher Scientific). The dissociated cells (2 × 10^5^ cells per reaction) were centrifuged at 200 × *g* for 5 min and resuspended in P3 primary cell nucleofector solution (Lonza) prior to the addition of 5 μL RNP solution, 2 μL of 100 μM ssODN (for CRISPR correction), and 4 μL electroporation enhancer. The iPSC-RNP mix was nucleofected using a P3 Primary Cell 4D-Nucleofector X Kit S (Lonza) using program CA-137. iPSC clones were expanded, and individual clones were mechanically isolated and plated into individual wells of a Geltrex-coated 12-well plate. Clonal iPSC lines were expanded and screened for the desired edit by Sanger sequencing (Source Bioscience).

### RPE differentiation

iPSCs were cultured to 90%–100% confluency on Geltrex-coated plates (Thermo Fisher Scientific) and then directed to differentiate into RPE cells following the protocol described by Regent et al.^20^ The RPE differentiation medium comprised high-glucose Dulbecco’s modified Eagle’s medium (Thermo Fisher Scientific), supplemented with 50 μM β-mercaptoethanol (Thermo Fisher Scientific), 1× minimum essential medium-nonessential amino acids (Thermo Fisher Scientific), and knockout serum replacement (KSR, Thermo Fisher Scientific) at 20% from days 0 to 50, which was reduced to 4% after passage 1. Additional supplements included 10 mM nicotinamide (Sigma-Aldrich) from days 0–7, 100 ng/mL activin A (PeproTech, London, UK) from days 7–14, and 3 μM CHIR99021 (Cell Guidance Systems, Cambridge, UK) until passage 1. Media changes were carried out every 2 to 3 days. Around day 50, cells were treated with TrypLE Express Reagent (Thermo Fisher Scientific) at 37°C for 10 min. Visibly pigmented patches were manually isolated, and cells were further incubated for up to 1 h with TrypLE Express to ensure dissociation. Finally, cells were seeded onto Geltrex-coated plates at a density of 100,000 cells per cm^2^ for subsequent experiments.

### Antisense oligonucleotide gymnosis

Gymnotic ASO experiments took place by adding the desired ASO concentration into the media once a week. Half media changes were carried out 3 days later, with no ASO added.

### Immunostaining

iRPE monolayers grown in 12-well plates were washed with DPBS (Thermo Fisher Scientific) and were carefully removed using a cell scraper and transferred to bijou tubes, where they were fixed in 4% paraformaldehyde (Thermo Fisher Scientific) for 10 min at RT. The iRPE was washed 3× in DPBS, and following the last wash, were incubated in 30% sucrose at 4°C overnight. iRPE monolayers were vertically embedded in OCT compound (CellPath, Newtown, UK) and frozen on dry ice. Blocks were stored at −80°C prior to sectioning at 14 μm onto charged slides using a Leica CM1850 cryostat. Cells grown on chamber slides were washed with PBS (Thermo Fisher Scientific) and fixed in 4% paraformaldehyde (Thermo Fisher Scientific) for 10 min at RT. Cells grown in chamber slides and vertically embedded iRPE were permeabilized using 0.1% Triton X-100 (Sigma-Aldrich) for 15 min before being blocked using a solution containing 0.3% bovine serum albumin (BSA, Sigma-Aldrich) and 10% FBS (Thermo Fisher Scientific) for 1 h at RT. Primary antibodies (Table S6) were diluted at the appropriate concentration in blocking solution and incubated for 1 h at RT. Samples were washed 3× with PBS (Thermo Fisher Scientific) and subsequently incubated with secondary antibodies (Table S7) that reacted against the primary antibody species for 1 h at RT, before being washed 3× with PBS. For BODIPY 493/503 (Thermo Fisher Scientific) staining, samples were incubated for 15 min using a 10 mM BODIPY stock that was diluted 1:1,000 following 2× washes with PBS. All samples were incubated with 4,6-diamidino-2-phenylindole (DAPI; 2 mg/mL) (Invitrogen, Thermo Fisher Scientific) in PBS for 5 min, then washed 3× with PBS. Slides were mounted in fluorescence mounting media (Dako, Agilent, CA, USA). All images were acquired using a Zeiss LSM700 confocal microscope using identical confocal settings (laser power, detector gain, pinhole size) for every condition (ISOCON, NT R345W, ASO1.1 treated R345W, and, where applicable, ISOKO) within an experiment. Acquisition parameters were established using control samples (ISOCON), and images were acquired below saturation. Quantification was performed on raw images without post-acquisition intensity adjustment. Images were prepared using ImageJ, Adobe Photoshop, and Adobe Illustrator.

### Fluorescence quantification

Single-channel confocal images (PNG format) were analyzed using ImageJ (NIH). Images were converted to 8-bit grayscale prior to analysis (image > type > 8-bit). A global intensity threshold was applied to segment the fluorescent signal from the background (image > adjust > threshold). The same threshold values were used for all images within an experimental set to ensure consistency and avoid bias. Regions of interest corresponding to the threshold signal were analyzed using the Analyze Particles/Measure function, and fluorescence was quantified as integrated density (sum of pixel intensities within the threshold area). For each image, the integrated density was recorded and used for statistical analysis.

### Western blotting

iRPE was washed with PBS and lysed on ice for 30 min in 80–120 μL cold RIPA buffer (50 mM Tris-HCl [pH 7.5], 150 mM NaCl, 1 mM EDTA, 1% NP-40, 0.5% sodium deoxycholate, and 0.1% SDS), supplemented with 2% protease inhibitor cocktail (Sigma-Aldrich). The samples were briefly sonicated before centrifuging at 4°C for 1 min at 13,000 rpm. Protein concentration was quantified using the Pierce BCA Protein Assay kit (Thermo Fisher Scientific) as per the manufacturer’s instructions. Protein samples (20 μg) were mixed with 5× sample buffer (250 mM Tris-HCI [pH 6.8], 50% v/v glycerol, 10% w/v SDS, and 525 mM freshly added DTT) before being heated at 100°C for 3 min to facilitate protein denaturation. Samples were resolved on precast 4%–20% gradient gels (Bio-Rad, Watford, UK) at 100 V until the dye reached the end of the gel. Pageruler Plus protein ladder (Thermo Fisher Scientific) was used to provide a reference for protein size. Proteins were transferred to a nitrocellulose membrane by wet transfer for 90 min, using standard protocols. Membranes were blocked overnight at 4°C in 5% skimmed milk powder in PBS + 0.1% Tween 20 (PBS-T), then incubated for 1 h in primary antibody, before undergoing 3 × 10-min washes with PBS-T. The membranes were then incubated in species-specific HRP-conjugated secondary antibody for 1 h before undergoing 3 × 10-min washes with PBS-T. All antibodies are listed in Tables S6 and S7 (Table S6). Membranes were developed using Clarity MAX Western ECL (Bio-Rad) for 5 min. Membranes were imaged using a ChemiDoc MP Imaging System (Bio-Rad). Images were formatted using Image Lab (Bio-Rad). Three independent protein isolates were used per condition.

### Subcellular fractionation

iRPE were washed with PBS and lysed on ice in 80–120 μL cold RIPA buffer, supplemented with 2% protease inhibitor cocktail (Sigma-Aldrich), as described above. The soluble fraction (cell lysate) was removed, and the cell pellet was resuspended in an equal volume (80–120 μL) of 1× sample treatment buffer. Samples were heated at 100°C for 3 min before resolving on precast 4%–20% gradient gels (Bio-Rad), and western blotting was carried out as described above. Nitrocellulose membranes were stained with Ponceau S staining solution (Thermo Fisher Scientific) prior to western blotting. Three independent protein isolates were used per condition (*n* = 3). Protein band intensities were quantified by densitometry using ImageJ (NIH). For each blot, the same rectangular region of interest was used to measure the integrated density for all bands. Target protein levels were normalized to the corresponding loading control—GAPDH (cell lysates) or Ponceau S (pellet fractions)—and data were expressed relative to the control condition (ISOCON iRPE).

### Photoreceptor outer segment phagocytosis assays

Porcine POS were isolated as previously described.^37^ In brief, neuroretina was removed from porcine eyes into homogenizing solution (20% [w/v] sucrose, 20 mM Tris acetate [pH 7.2], 2 mM magnesium chloride, 10 mM glucose, and 5 mM taurine), shaken vigorously for 2 min, filtered, and centrifuged against a continuous sucrose gradient for 1 h at 25,000 rpm in a Beckman SW-27 using an SW32-Ti swing rotor (Brea, CA) at 4°C. The POS-containing fraction (orange band) was washed and resuspended in DMEM containing 10% FBS. Prior to phagocytosis assays, POS were sonicated for 10 min on ice and incubated on ice for a further 5 min, and iRPE cells cultured on transwell filters were incubated with sonicated POS (200 μg/mL) for 4 h at 37°C. Cells were washed extensively (5×) in DPBS and then processed for immunocytochemistry with RET-P1 primary antibody (Table S6), as described above, to detect internalized rhodopsin. To quantify POS phagocytosis, the percentage of cells positive for internalized POS was counted.

### TEM

iRPE cells cultured on transwell filters were fixed in EM fixative (2% glutaraldehyde and 2% paraformaldehyde in 0.1 M sodium cacodylate buffer [pH 7.4]) for 30 min at RT and post-fixed with 1% osmium tetroxide/1.5% potassium ferricyanide in 0.1 M sodium cacodylate buffer for 1 h at 4°C. Cells were stained with 2% uranyl acetate replacement solution (UA Zero) for 1 h, dehydrated through an ethanol series, and infiltrated with TAAB-812 epoxy resin (TAAB Laboratories, Aldermaston, UK) prior to polymerization overnight at 60°C. Ultrathin sections were imaged on a JEOL 1400Plus EM (JEOL Ltd, Tokyo, Japan) fitted with an Advanced Microscopy Technologies (AMT) NanoSprint12 camera (AMT Imaging Direct, Woburn, MA, USA). Quantitation of the accumulations between the basal membrane and underlying transwell membrane was conducted manually and blind to the condition: ISOCON iRPE, 40 cells (∼10 μm/cell); R345W iRPE, 25 cells (∼7.8 μm/cell); and ASO1.1-treated R345W iRPE, 22 cells (∼9.7 μm/cell).

### Processing of DNA and RNA from iRPE

Total gDNA from cell samples was extracted using the Wizard gDNA Purification kit (Promega) following the manufacturer’s guidelines. Total RNA from cell samples was extracted using the RNeasy Micro kit (Qiagen) following the manufacturer’s guidelines. cDNA was synthesized (Tetro cDNA synthesis, Bioline) using equal quantities of total RNA for each sample (1 μg) and carried out according to the manufacturer’s guidelines.

### qCR of iRPE samples

qPCR was used to determine the relative levels of *EFEMP1* gene expression (normalized to the housekeeping genes *ACTB* and *GAPDH*), by measuring the fluorescence emitted by DNA bound to the intercalating dye SYBR green. qPCR reactions were assembled using 2× LabTaq Green Hi Rox Master Mix (Labtech, Heathfield, UK) and conducted with an Applied Biosystems QuantStudio 6 Flex real-time PCR system.

### NGS

Universal tagged primers for MiSeq (Illumina) high-throughput sequencing (HTS) are listed in Table S5. PCR amplification was carried out using High-Fidelity 2× master mix (NEB). The products were gel extracted (Monarch DNA Gel Extraction Kit, NEB), and 300–500 ng of product was used for subsequent steps. Processing of the samples using MiSeq Reagent Nano kit v2 was carried out at the UCL Cancer Institute CAGE Facility.

### Allele quantification from MiSeq sequencing and integration with qPCR expression data

FASTQ files generated from targeted Illumina MiSeq sequencing of the EFEMP1 amplicon were analyzed using a custom Python script (Table S8). For each sample, sequencing files were parsed, and the number of reads containing allele-specific sequence motifs was counted. Reads containing the sequence ATGCCGGGAGGA were classified as WT, whereas reads containing ATGCTGGGAGGA were classified as the R345W variant. These sequences encompass the single nucleotide substitution responsible for the R345W mutation and uniquely distinguish the two alleles. The total number of WT and R345W reads was determined for each sample. Allele proportions were calculated from the total informative reads. Relative EFEMP1 expression (RQ) values were obtained from the qPCR analysis described previously. Allele-specific expression was estimated by multiplying the RQ value for each sample by the corresponding MiSeq-derived allele proportion. This approach allowed partitioning of total EFEMP1 expression into estimated contributions from the WT and R345W alleles under each experimental condition.

### Data visualization and statistical analysis

Statistical analyses were carried out using RStudio (R v4.2.1). One-way ANOVAs were carried out using the base R aov function and post hoc analyses using the RStudio package rstatix (v0.7.2). All graphs presented were generated using the RStudio package using the RStudio package ggplot2 (v3.4.1).

## Data and code availability

The data related to this study are fully documented in the paper or in the supplemental materials. The raw MiSeq FASTQ files have been deposited in Zenodo (https://doi.org/10.5281/zenodo.18633265 and https://doi.org/10.5281/zenodo.18633265).

## Acknowledgments

The authors gratefully acknowledge the funding from the Macular Society (20-RG-04 to J.v.d.S. (PI), A.-J.F.C. (Co-I), M.E.C. (Co-I), and M.M. (Co-I)). This research was supported by the National Institute for Health and Care Research Biomedical Research Centre at Moorfields Eye Hospital and UCL Institute of Ophthalmology and the Wellcome Trust (099173/Z/12/Z to M.M. and 205041/Z/16/Z to M.E.C.). pmScarlet_C1 was a gift from Dorus Gadella (Addgene plasmid # 85042). miRNA 302/367 plasmid was a gift from Dr. J.A. Thomson, Regenerative Biology, Morgridge Institute for Research, Madison, Wisconsin, USA (Howden et al., 2015). pCXLE-hOCT3/4-shp53-F (Addgene, plasmid #27077), pCXLEhSK (Addgene, plasmid #27078), and pCXLEhUL (Addgene, plasmid #27080) were gifts from Shinya Yamanaka. We thank the Cancer Genomics Engineering (CAGE) Facility, UCL Cancer Institute, for MiSeq preparation and sequencing. The CAGE Facility is supported by the BRC, Welton Foundation, and in part by the Cancer Research UK – UCL Center.

## Author contributions

Conceptualization, J.v.d.S.; data curation, F.O.R., E.R.E., and J.v.d.S.; formal analysis, F.O.R., B.S.-P., E.R.E., and J.v.d.S.; funding acquisition, J.v.d.S., A-J.F.C., M.E.C., and M.M.; investigation, F.O.R., B.S.-P., and E.R.E.; methodology, F.O.R., B.S.-P., E.R.E., J.C.C.-S., and J.v.d.S.; project administration, J.v.d.S.; resources, N.A., A.R.W., T.A.C.d.G., A.-J.F.C., M.E.C., and M.M.; supervision, J.v.d.S.; software, F.O.R.; validation, F.O.R., B.S.-P., E.R.E., and J.v.d.S.; visualization; F.O.R., B.S.-P., E.R.E., and J.v.d.S.; writing – original draft; F.O.R. and J.v.d.S.; writing – review and editing, F.O.R., B.S.-P., E.R.E., N.A., A.R.W., A.-J.F.C., M.E.C., M.M., and J.v.d.S.

## Declaration of interests

An international patent application (PCT/GB2024/052082) related to this work has been filed in collaboration with UCL Business Ltd. (UCLB). J.v.d.S., F.O.R., and B.S.-P. are listed as inventors on this application.

## Supplemental information

Document S1. Figures S1–S3 and Tables S1–S8

Document S2. Article plus supplemental information
