# Supplementary material for: Antisense oligonucleotide allele-specific targeting of EFEMP1 in a patient-derived model of Doyne honeycomb retinal dystrophy
Source: Mol Ther Nucleic Acids. 2026 May 19;37(2):102955. doi: 10.1016/j.omtn.2026.102955 (PMC13266002; doi:10.1016/j.omtn.2026.102955)
Supplement: Document S1. Figures S1–S3 and Tables S1–S8 [file mmc1.pdf]

OMTN, Volume *37*

Supplemental information

Antisense oligonucleotide allele-specific targeting of EFEMP1 in a patient-derived model of Doyne honeycomb retinal dystrophy

Farah O. Rezek, Beatriz Sanchez-Pintado, Emily R. Eden, Julio C. Corral-Serrano, Nancy Aychoua, Andrew R. Webster, Thales A.C. de Guimarães, Amanda-Jayne F. Carr, Michel Michaelides, Michael E. Cheetham, and Jacqueline van der Spuy

SUPPLEMENTAL INFORMATION

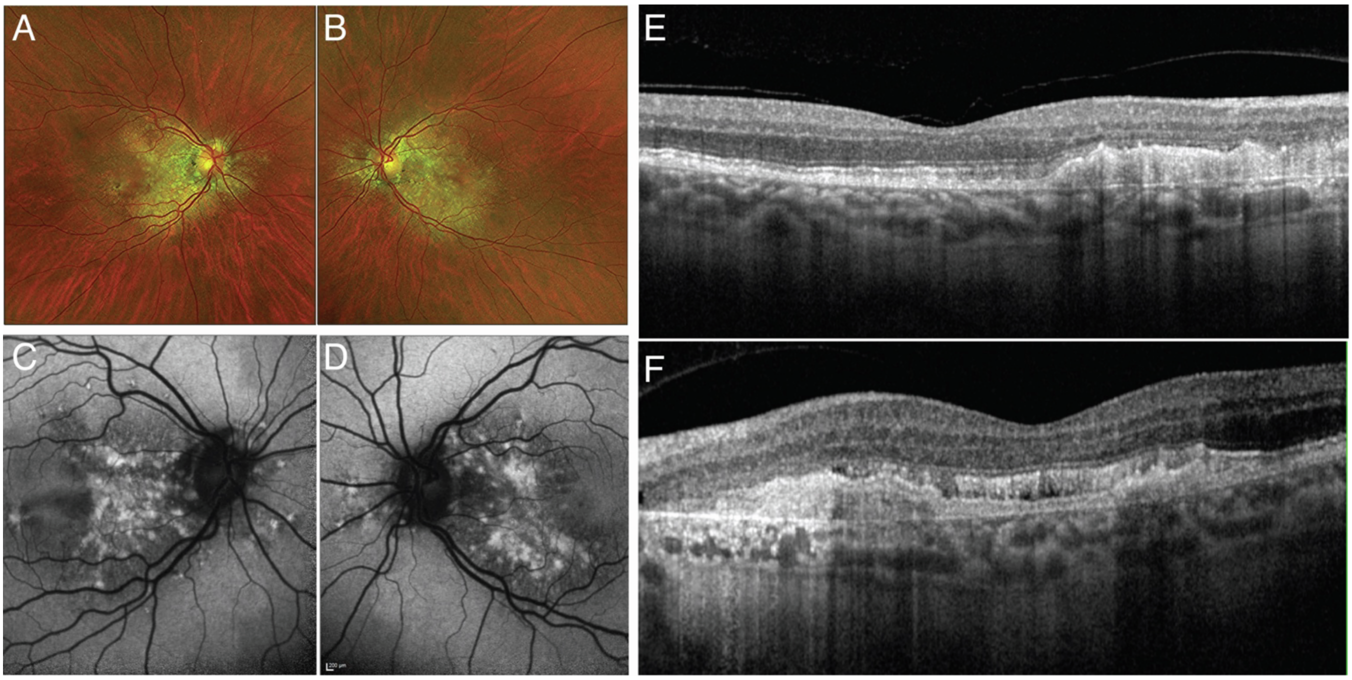

Figure S1: Clinical features of DHRD patient at 60 years of age.
Related to Figure 1.
(A) Ultra-widefield fundus pseudocolour image (Optos scanning laser ophthalmoscope) of right eye.
(B) Ultra-widefield fundus pseudocolour image (Optos scanning laser ophthalmoscope) of left eye.
(C) Autofluorescence image of right eye (HEYEX blue light AF).
(D) Autofluorescence image of left eye (HEYEX blue light AF).
(E) Heidelberg Spectralis OCT image of right eye.
(F) Heidelberg Spectralis OCT image of left eye.

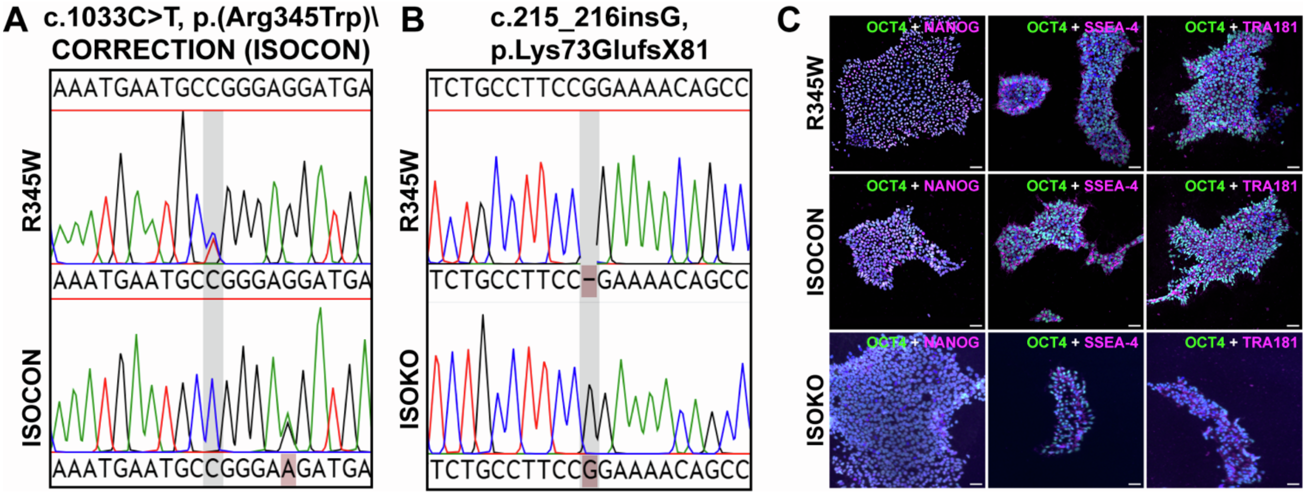

Figure S2: CRISPR-Cas9 genome editing of EFEMP1 isogenic control and knockout iPSC
Related to Figure 1.
(A) Sanger sequencing chromatogram of gDNA from patient-derived R345W iPSC (top) and isogenic control (ISOCON) iPSC (bottom) following CRISPR-Cas9 HDR to correct *EFEMP1* c.1033C>T (grey bar). The synonymous change in the PAM (AGG-AAG) is coloured pink.
(B) Sanger sequencing chromatogram of gDNA from patient-derived R345W iPSC (top) and isogenic knockout (ISOKO) iPSC (bottom) following CRISPR-Cas9 NHEJ to knockout the *EFEMP1* gene. The grey bar highlights the insertion of a single homozygous 'G' at c.125.
(C) Characterization of ISOCON and IOSKO iPSC clones. Immunocytochemistry analysis of pluripotency markers Oct4, Tra-1-81, Nanog and SSEA-4 in patient-derived R345W, ISOCON and ISOKO iPSC. Scale bars, 10 μm.

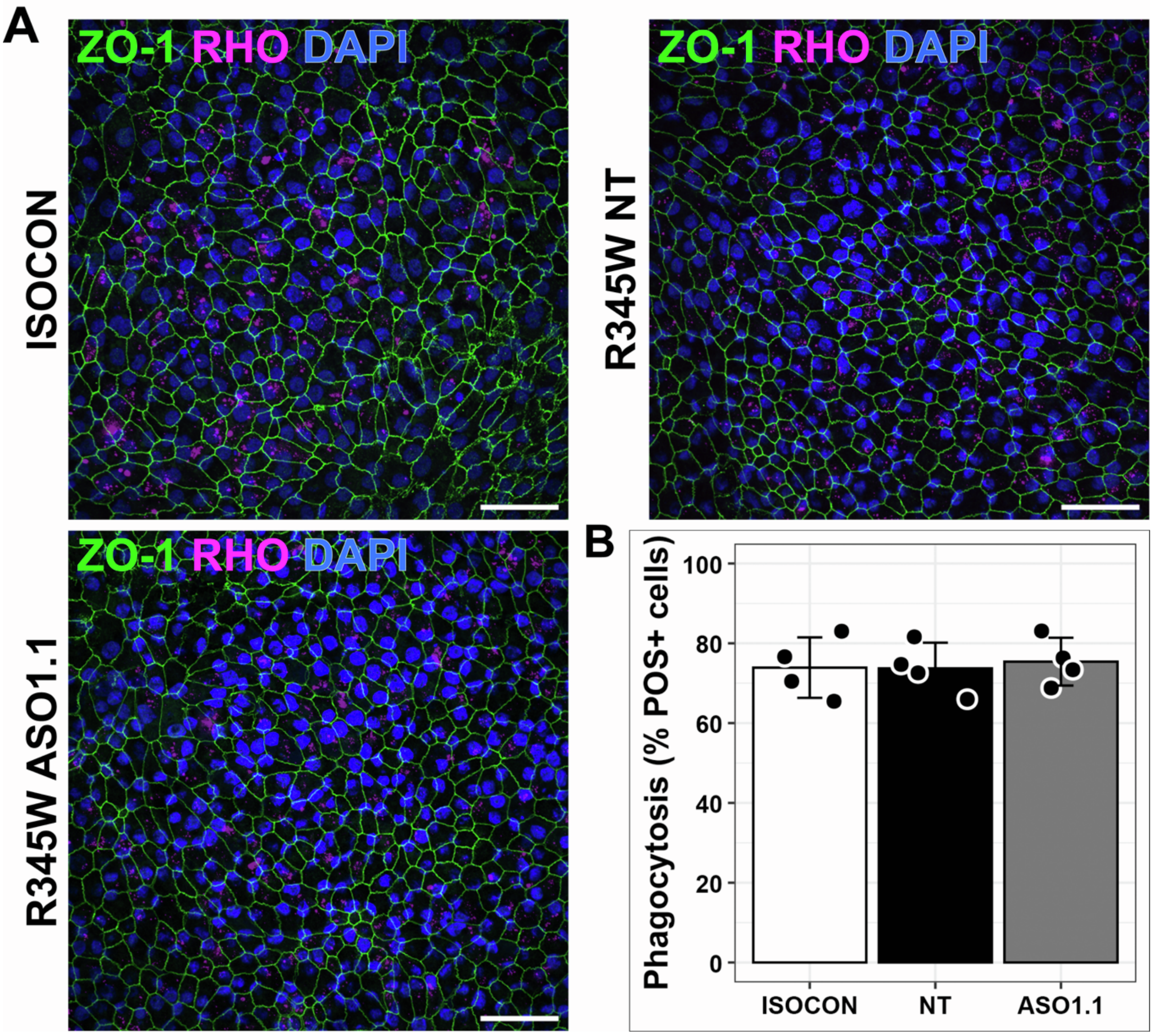

Figure S3: Photoreceptor outer segment phagocytosis
Related to Figure 7.
(A) IF of photoreceptor outer segment (POS) phagocytosis in ISOCON, R345W NT and R345W ASO1.1 treated iRPE showing POS (magenta) and tight junction marker ZO-1 (green). Nuclei (blue) are labelled with DAPI. Scale bars, 50 µm.
(B) Quantitation of POS phagocytosis in ISOCON, R345W NT and R345W ASO1.1 treated iRPE. N = 4 images. Statistical significance was determined by one way ANOVA followed by post-hoc Tukey's (HSD) test where *, ** and *** denotes a p value <0.05, 0.01 and 0.005 respectively. ns = not significant. Bars represent mean ± SD.

SUPPLEMENTAL TABLES

Table S1: CRISPR Correction of c.1033C>T: crRNA and ssODN sequences

**Desired change**	**crRNA (5'-3')**	**GC%**	**ssODN (5'-3')**
c.1033T>C	GACCACAAATGAATGCTGGG	50%	TTCTCTGGTGTTAGAATGTAGG GATCTTGACAAGGATTTCGTGG ATAACAACGGAAGCCGCCATG ATAATTCCAACACATTTCATCT**T** CCCGGCATTCATTAGTGGTCT CACACTCATTTATATCTG

cRNA: Target residue (T): large font size. **ssODN**: Complementary to cRNA (underlined). Target repair (G): large font size. Synonymous PAM change (T): bold

Table S2: CRISPR *EFEMP1* Knockout: cRNA Sequence

**Resulting change**	**cRNA (5'-3')**	**GC%**
c.215_216insG, p.Lys73GlufsX81	TCCGAAAACAGCCCAGATTA	45%

Table S3: Cas-OFFinder Analysis of *In Silico* Predicted Off Targets

Guide purpose	Site of potential off-target editing	Sequence of potential binding site	Mismatches
CRISPR Correction of c.1033C>T	chr6:51,829,169-51,829,191	crRNA: GACCACAAATGAATGCTGGG DNA: tAgCACAAATcAATGCTGGG-TGG	3
CRISPR Correction of c.1033C>T	chr16:64,364,348-64,364,370	crRNA: GACCACAAATGAATGCTGGG DNA: GAtCACAAgTGcATGCTGGG-AGG	3
CRISPR Correction of c.1033C>T	chr8:144,720,814-144,720,836	crRNA: GACCACAAATGAATGCTGGG DNA: GAgCACAAAgGAATtCTGGG-TGG	3
CRISPR Correction of c.1033C>T	chr10:29,247,464-29,247,486	crRNA: GACCACAAATGAATGCTGGG DNA: GACaAgAAATGtATGCTGGG-AGG	3
CRISPR Correction of c.1033C>T	chr1:211,488,887-211,488,909	crRNA: GACCACAAATGAATGCTGGG DNA: GACCtCAgATGcATGCTGGG-TGG	3

CRISPR Correction of c.1033C>T	chr5:155,412,417-155,412,439	crRNA: GACCACAAATGAATGCTGGG DNA: agaCACAAAaGAATGCTGGG-CGG	4
CRISPR Correction of c.1033C>T	chr3:8,212,410-8,212,432	crRNA: GACCACAAATGAATGCTGGG DNA: ttCaACAcATGAATGCTGGG-GGG	4
CRISPR Correction of c.1033C>T	chr10:73,553,379-73,553,401	crRNA: GACCACAAATGAATGCTGGG DNA: ctCCACAgATGcATGCTGGG-CGG	4
CRISPR Correction of c.1033C>T	chr6:10,342,654-10,342,676	crRNA: GACCACAAATGAATGCTGGG DNA: ttCCACAcATGAATtCTGGG-GGG	4
CRISPR Correction of c.1033C>T	chr14:58,766,075-58,766,097	crRNA: GACCACAAATGAATGCTGGG DNA: cAaggCAAATGAATGCTGGG-AGG	4
EFEMP1 knockout	chr4:72,743,031-72,743,053	crRNA: TCCGAAAACAGCCCAGATTA DNA: TCaGAAAcCAGCtCAGATTA-TGG	3
EFEMP1 knockout	chr6:135,177,560-135,177,582	crRNA: TCCGAAAACAGCCCAGATTA DNA: ggtGAAAACAGaCCAGATTA-GGG	4
EFEMP1 knockout	chr4:93,297,559-93,297,581	crRNA: TCCGAAAACAGCCCAGATTA DNA: atCtAAAAaAGCCCAGATTA-GGG	4
EFEMP1 knockout	chr19:17,948,974-17,948,996	crRNA: TCCGAAAACAGCCCAGATTA DNA: aaCGAcAAtAGCCCAGATTA-GGG	4
EFEMP1 knockout	chr14:76,683,368-76,683,390	crRNA: TCCGAAAACAGCCCAGATTA DNA: gCtaAAtACAGCCCAGATTA-TGG	4
EFEMP1 knockout	chr3:170,532,773-170,532,795	crRNA: TCCGAAAACAGCCCAGATTA	4

		DNA: aCaGAAAAaAGCCCtGATTA-AGG	
EFEMP1 knockout	chr8:134,919,582-134,919,604	crRNA: TCCGAAAACAGCCCAGATTA DNA: aCCaAgAACAtCCCAGATTA-GGG	4
EFEMP1 knockout	chr1:52,983,771-52,983,793	crRNA: TCCGAAAACAGCCCAGATTA DNA: aCCcAAgAaAGCCCAGATTA-TGG	4
EFEMP1 knockout	chr9:119,373,155-119,373,177	crRNA: TCCGAAAACAGCCCAGATTA DNA: aCCcAAAgCAcCCCAGATTA-AGG	4
EFEMP1 knockout	chr2:163,975,413-163,975,435	crRNA: TCCGAAAACAGCCCAGATTA DNA: TtCtAAAACAtgCCAGATTA-AGG	4

Table S4: ASO Sequences

**Name**	**Sequence (5'-3')**	**Chemistry**
CTRL ASO	CACCCCCATTCTTCAGCC	PS backbone; 2'-O-MOE
ASO1	UCAUCCTCCCAGCAUUCA	PS backbone; 2'-O-MOE; LNA*
ASO2	UCAUCCTCCCAGCAUUCA	PS backbone; 2'-O-MOE, LNA**
ASO3	AUCCUCCCAGCATUCAUU	PS backbone; 2'-O-MOE; LNA*
ASO4	AUCCUCCCAGCATUCAUU	PS backbone; 2'-O-MOE; LNA**
ASO1.1	CATCCTCCCAGCATTC	PS backbone; 2'-O-MOE; LNA*
ASO1.2	TCATCCTCCCAGCAT	
ASO1.3	ATCCTCCCAGCATTC	
ASO1.4	ATCCTCCCAGCATT	

PS, phosphorothioate; 2'-O-MOE, 2'-O-methoxyethyl; LNA, single linked nucleic acid; LNA**, two linked nucleic acids.*

Table S5: Primers

**EFEMP1 PCR Primers**	
**Name**	**Sequence (5'-3')**
EFEMP1-201_1F (Forward)	TGTGCTGTGCAAGGAACTCT
EFEMP1-201_2F (Forward)	GCTGTGCAAGGAACTCTGCT

EFEMP1-201_1R (Reverse)	TTGGCTGACTTAAATGCCTGT
**EFEMP1 Sequencing Primers**	
EFEMP1 c.1033C>T cDNA F (Forward)	TGCAGAACCTCAAGCTACCTGTGTC
EFEMP1 c.1033C>T cDNA R (Reverse)	GGGCAAACACATCGGTTCTCTGG

**qPCR Primers**		
**Name**	**Forward (5'-3')**	**Reverse (5'-3')**
MScarlet	GAGTTCATGCGGTTCAAGGT	ACATGAACTGAGGGGACAGG
3xFLAG	GTATAGGGACCTTCCGCACA	GGAGGGGTCACAGGGATG
EFEMP1	GTTTCCTGCTGAGGCTGTTC	CAGGACACCGAAGAAACCAT
GAPDH	CCCCACCACACTGAATCTCC	GGTACTTTATTGATGGTACATGACAAG
ACTB	CCAACCGCGAGAAGATGA	CCAGAGGCGTACAGGGATAG

**High Throughput Sequencing (HTS) Primers**			
**Forward MiSeq primer (5'-3')**	**Reverse MiSeq primer (5'-3')**	**Amplicon Size**	
		**bp w/o tags**	**bp w/ tags**
**TCGTCGGCAGCGTCAGATGTGTATAAGAGACAG**TGCAGAACCTCAAGCTACCTGTGTC	**GTCTCGTGGGCTCGGAGATGTGTATAAGAGACAG**GGGCAAACACATCGGTTCTCTGG	248	315
**TCGTCGGCAGCGTCAGATGTGTATAAGAGACAG**CCTTCCTTGCAAACAGAATCTGCC	**GTCTCGTGGGCTCGGAGATGTGTATAAGAGACAG**GCAGTTTGGCTTGGTAAGACCAG	276	343

Table S6: Primary Antibodies

**Antigen**	**Host**	**Supplier**	**Catalogue number**	**Dilution**
ZO-1	Rabbit	Thermo Fisher Scientific	61-7300	1:1000 (WB)<br>1:200 (IF)
PMEL	Mouse	Agilent	M0634	1:50 (IF)
MERTK	Rabbit	Abcam	ab52968	1:500 (WB)<br>1:200 (IF)
EZRIN	Mouse	Thermo Fisher Scientific	MA5-13862	1:500 (WB)<br>1:200 (IF)
GAPDH	Mouse	Proteintech	60004-1-Ig	1:10,000 (WB)
Collagen IV	Goat	Bio-techne	NBP1-26549	1:200 (IF)
APOE	Mouse	Bio-techne	NB110-60531	1:200 (IF)

Fibulin 3	Mouse	Santa Cruz Biotechnology	sc-33722	1:250 (IF)
RET-P1	Mouse	Santa Cruz.	sc-57433	1:200
OCT4	Rabbit	Abcam	Ab19857	1:1000 (IF)
NANOG	Mouse	Invitrogen	MA1-017	1:200 (IF)
SSEA4	Mouse	Cell Signalling Technologies	4755	1:500 (IF)
TRA181	Mouse	Cell Signalling Technologies	4745	1:1000 (IF)

Table S7: Secondary Antibodies

Antigen	Host	Fluorophore	Supplier	Catalogue number	Dilution
Rabbit IgG	Donkey	488	Invitrogen	A32790	1:1000
Mouse IgG	Donkey	555	Invitrogen	A31571	1:1000
Goat IgG	Donkey	488	Invitrogen	A-21432	1:1000
Rabbit-HRP	Goat	-	Invitrogen	31460	1:30,000
Mouse-HRP	Goat	-	Invitrogen	31430	1:30,000

Table S8. Python script for HTS data analysis

```
import os
sorted(os.listdir('C:\filepath'))
for info in sorted(os.listdir('C:\filepath')):
    domain = os.path.abspath('C:\filepath')
    info = os.path.join(domain, info)
    info = open(info, 'r')
    a = info.read()
    info.close()
    WT = a.count('ATGCCGGGAGGA')
    R345W= a.count('ATGCTGGGAGGA')
    print('WT: ', WT)
    print('R345W: ', R345W
```
